# Increased hydrogen and ethanol production in transformants of the filamentous cyanobacterium *Phormidium lacuna*

**DOI:** 10.1007/s00203-026-04932-4

**Published:** 2026-05-30

**Authors:** Nina Spohrer, Florian Reinicke, Stefan Malik, Racha Tarakji, Nora Weber, Tilman Lamparter

**Affiliations:** Joseph Gottlieb Kölreuter Institute for Plant Sciences (JKIP), Karlsruhe Institute of Technology (KIT), Fritz-Haber-Weg 4, 76131 Karlsruhe, Germany

**Keywords:** Natural transformation, Homologous recombination, Cyanobacteria, Oscillatoriales, Marine organism, Biohydrogen, Bioethanol, Protein expression, Promoter engineering

## Abstract

Research on biofuel production by cyanobacteria has largely focused on unicellular species. To date, there are no reports on engineered filamentous cyanobacteria for bioethanol or biohydrogen production. However, biofilm formation in filamentous cyanobacteria could provide an advantage for biotechnological applications. We used natural transformation and homologous recombination to establish recombinant expression at the *psaD* locus of the filamentous cyanobacterium *Phormidium lacuna*. A similar approach has already been successful in the unicellular cyanobacterium *Synechocystis* sp. PCC 6803 (Appel et al. 2020). Fusion of the NiFe hydrogenase subunits HoxY/HoxH from *Microcystis aeruginosa* to the PSI subunit PsaD increased hydrogen production five-fold under light in the presence of DCMU, although dark fermentative hydrogen levels remained higher. Structural modeling indicates that electron transfer may be limited by the FeS cluster distance, suggesting opportunities for further optimization. Using sfGFP fluorescence, we showed that the *psaD* promoter drives strong expression when the gene is positioned 3′ of the *psaD* gene or expressed as a PsaD–sfGFP fusion protein. The *psaD* promoter was then used for expression of pyruvate decarboxylase and alcohol dehydrogenase, resulting in ethanol production of 0.2‰ (v/v) and outperforming the kanamycin promoter used in previous unsuccessful trials. These results demonstrate that *P. lacuna* is a robust host for biohydrogen and bioethanol production and validate the *psaD* promoter as a strong regulatory element for filamentous cyanobacteria in biotechnological applications.

**Supplementary Information:**

The online version contains supplementary material available at 10.1007/s00203-026-04932-4.

## Introduction

Cyanobacteria are a subgroup of the bacterial domain that originated approximately 3.5 billion years ago. Their unique characteristic, compared to other bacteria, is their ability to perform oxygenic photosynthesis, in which two photosystems, PSII and PSI, drive a light-driven electron transfer cascade from water to ferredoxin and NADP⁺. This electron transfer forms the basis for cyanobacterial growth, which can rely solely on sunlight as an energy source and does not require additional organic energy inputs (Dismukes et al. 2008). Cyanobacteria contribute approximately 25–50% of marine biomass production, whereas global terrestrial biomass production is dominated by eukaryotic land plants. For sunlight-based biotechnological production, for example of biofuels, aquatic cyanobacterial species may have the advantage of higher areal growth rates (Dismukes et al. 2008).

Bioethanol is currently produced from crop plants such as corn, sugar beet, or sugarcane, but ongoing research aims to establish bioethanol production in cyanobacteria. The goal is to enable direct ethanol production in cyanobacteria through genetic engineering (Angermayr et al. 2009; Machado et al. 2012). Cyanobacteria do not possess a native ethanol production pathway; however, recombinant expression of pyruvate decarboxylase and alcohol dehydrogenase from *Zymomonas mobilis* in cyanobacteria has resulted in ethanol production, albeit at still low levels (Deng et al. 1999; Dexter et al. 2009; Velmurugan et al. 2020). A commercial application of bioethanol production by cyanobacteria by the Algenol company in Florida was not successful because production rates remained too low. To achieve commercial viability, more basic research regarding optimal strains, metabolic engineering, and bioreactor design is required.

Another biofuel that could be produced by cyanobacteria is hydrogen. Cyanobacteria can produce hydrogen naturally through the enzymatic activity of nitrogenases and hydrogenases (Madamwar et al. 2000). Nitrogenases incorporate molecular nitrogen (N₂) into organic molecules, a process restricted to prokaryotes. During nitrogen fixation, hydrogen is released as a byproduct. The uptake hydrogenase of cyanobacteria can consume some of this hydrogen to recover stored energy. The bidirectional hydrogenase can either consume or produce hydrogen to balance the cellular redox state. Both types of hydrogenases belong to the subgroup of NiFe hydrogenases, in contrast to the FeFe hydrogenases of green algae (Bothe et al. 2010). In general, hydrogenases and nitrogenases are sensitive to oxygen in vitro. The only known oxygen-insensitive hydrogenase is from the bacterium *Cupriavidus necator* (formerly *Ralstonia eutropha*) (Fritsch et al. 2011).

Natural production of biohydrogen from green algae or cyanobacteria, however, is too low for commercial applications (Zhang et al. 2023). To increase hydrogen production in cyanobacteria, ongoing research follows several strategies: (i) screening for cyanobacteria that produce high levels of hydrogen (Allahverdiyeva et al. 2010), (ii) genetic engineering, e.g., knockout of uptake hydrogenase (Masukawa et al. 2002), and (iii) recombinant expression of hydrogenases coupled to the electron transport chain of PSI.

Proof of principle for the latter approach was recently established by (Appel et al. 2020). This group engineered a fusion protein of the HoxY hydrogenase subunit of *Synechocystis* sp. PCC 6803 (hereafter *Synechocystis*) bidirectional hydrogenase with the PSI subunit PsaD. The fusion protein was expressed together with the HoxH hydrogenase subunit in a PsaD-deficient mutant of *Synechocystis*. The authors showed that the transformant produced ten times more hydrogen in the light with DCMU than the fermentative hydrogen production in the wild type. Electrons from the photosynthetic electron transport chain were transferred from PSI via PsaD to the hydrogenase. In the Hox-type NiFe hydrogenases of cyanobacteria, electrons flow via an iron-sulfur (FeS) cluster of the HoxY subunit to the active HoxH subunit, which contains iron and nickel ions for H₂ generation. Estimated distances between the FeS clusters of PSI and HoxY in the transformant were around 40 Å, which is too large for highly efficient electron transfer; shorter distances are likely required for optimal photohydrogen production.

According to their growth pattern, cyanobacteria can be divided into four major groups (Rippka et al. 1979): (i) unicellular species such as *Synechocystis*, (ii) filamentous species with heterocysts, (iii) filamentous species without heterocysts, and (iv) species with branched filaments. Most studies use unicellular species such as *Synechocystis* sp. PCC 6803, but there are only few bioengineering approaches using filamentous cyanobacteria (Tabakh et al. 2023; Mahanil et al. 2022). This is likely because genetic engineering has not been fully established for filamentous species, which are often more difficult to handle. However, filamentous cyanobacteria can offer industrial advantages. The filamentous *Spirulina* (*Arthrospira / Planktothrix*) *platensis* grows at large scale in open ponds for the production of food supplements (Abreu et al. 2023). Biofilm formation is often more pronounced in filamentous than in unicellular species. In a bioreactor where filaments attach to a surface, the medium of a strong ethanol producer could be replaced before ethanol reaches levels toxic to the cyanobacterium. Including filamentous species in biotechnological studies could enhance biofilm formation, increase biomass density, improve robustness under fluctuating environmental conditions, and potentially increase the productivity of desired compounds (Gross et al. 2015; Abreu et al. 2023).

In this study, we used *Phormidium lacuna* as a model filamentous cyanobacterium for bioethanol and biohydrogen production. *P. lacuna* was isolated from North Sea rock pools and tolerates temperatures up to 50 °C, salinities from 0.1 to 3.7× seawater, and high light intensities (Nies et al. 2017). *P. lacuna* is one of the few filamentous species for which natural transformation capability has been established (Nies et al. 2020; Tabakh et al. 2023), enabling reliable gene knockouts and heterologous protein expression (Weber et al. 2022; Lamparter et al. 2022). This positions it as a promising filamentous chassis for bioengineering.

We tested whether gene transformation in *P. lacuna* could replicate approaches previously performed in unicellular species. Ethanol production in *P. lacuna* had been attempted previously without success (Nies 2019). Here, we began with the hydrogen production approach mentioned above (Appel et al. 2020), constructing fusion proteins with PsaD and HoxY/H subunits from different cyanobacteria, and selected one construct for detailed study. Expression of the fusion protein was placed under the control of the endogenous PsaD promoter. To confirm promoter activity, we tested sfGFP expression in *P. lacuna* (Pédelacq et al. 2006). This success prompted us to test ethanol production under the control of the PsaD promoter.

Our results contribute to the increased use of filamentous cyanobacteria as tools for biofuel production and for the production of other valuable compounds using sunlight as an energy source. The ease of transformation suggests that *P. lacuna* can serve as a test system for metabolic improvements. These results could either be applied directly to *P. lacuna* cultures or transferred to other filamentous cyanobacteria such as *Arthrospira*, for which genetic transformation has also recently been established (Tabakh et al. 2023).

## Methods

### Culture growth and natural transformation

**Fig. 1:**
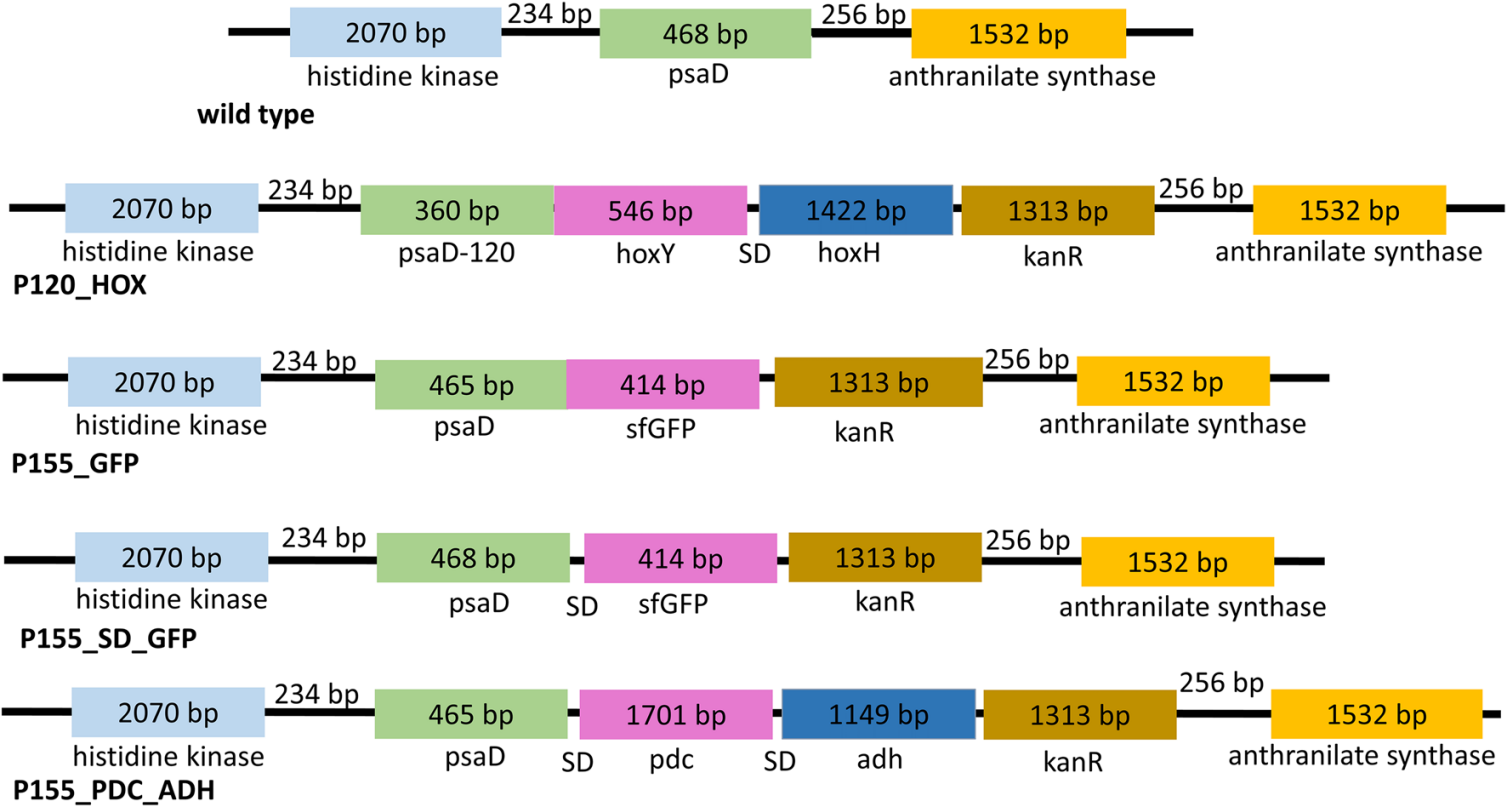
Gene arrangement of transformants after homologous integration. Light-blue and orange boxes show the regions 5’ and 3’ of the insertions. Inserted genes are presented by magenta, blue and brown boxes, the psaD coding region was also part of the transformation vector. The 5’ region used for homologous recombination extends over the proposed psaD promoter and a part of a histidine kinase gene, whereas the 3’ homologous region extends to an anthranilate synthase gene

The strain *Phormidium lacuna* HE10DO was used for the experiments. This strain was isolated from rock pools of the North Sea coast of the Helgoland island (Nies et al. 2017). Cultures are available at the DSMZ stock center (Braunschweig, Germany). For propagation and experiments, cultures were maintained in f/2 medium (Guillard et al. 1962) supplemented with 10x higher concentrations of phosphate and nitrate. Cultures were kept under constant agitation under white fluorescent light (40 µmol m⁻² s⁻¹ ). Cloning of transformation vectors is described in Supplementary document 1, the gene organization of transformants is summarized in Fig. 1. For natural transformation we followed the protocol of (Weber et al. 2022).

### Hydrogen measurements

For H_2_ measurements, *P. lacuna* wild type and the transformant P120_HOX were cultivated for three weeks. The cultures were then homogenized using a SilentCrusher M (Heidolph) at 10.000 rpm for three minutes. Filaments were collected by centrifugation at 6.000 g and 4 °C for 10 min, resuspended in f/2 medium and adjusted to an OD_750 nm_ = 4. Nine ml of this culture was poured into a 10 ml Headspace Screw Neck Vial (MACHEREY-NAGEL GmbH & Co. KG) that has an inner diameter of 18 mm. For DCMU treatments, 9 µL of a 100 mM DCMU solution were directly added to the cultures in the Headspace vials. Control measurements were made from headspace vials using growth medium without filaments, under otherwise identical conditions. To convert gas concentrations from vol % into µmol, precisely weighed quantities of aluminum powder were mixed with 9 ml of 0.1 M NaOH, transferred into headspace vials and processed in the same manner as described below for the experimental samples. The NaOH / Al reaction can be described by the formula.

$$ {\text{2Al + 2 NaOH + 6 H}}_{2} {\mathrm{O}} \to {\text{2 Na[Al(OH)}}_{{\mathrm{4}}} {\text{] + 3 H}}_{{\mathrm{2}}} $$

A ratio of 2.0 ± 0.4 µmol per vol % was estimated; we used a factor of 2 for conversions of vol % into µmol. The headspace vials were sealed with hydrogen-tight butyl/PTFE septa (MACHEREY-NAGEL GmbH & Co. KG) and incubated for 48 h either in darkness or in an LED light chamber with constant irradiation with strong white light (560 µmol m^-2^s^-1^) from the side without agitation. During the 48-h incubation the filaments settled and formed a layer of approximately 1 cm height at the bottom of the vial.

**Fig. 2:**
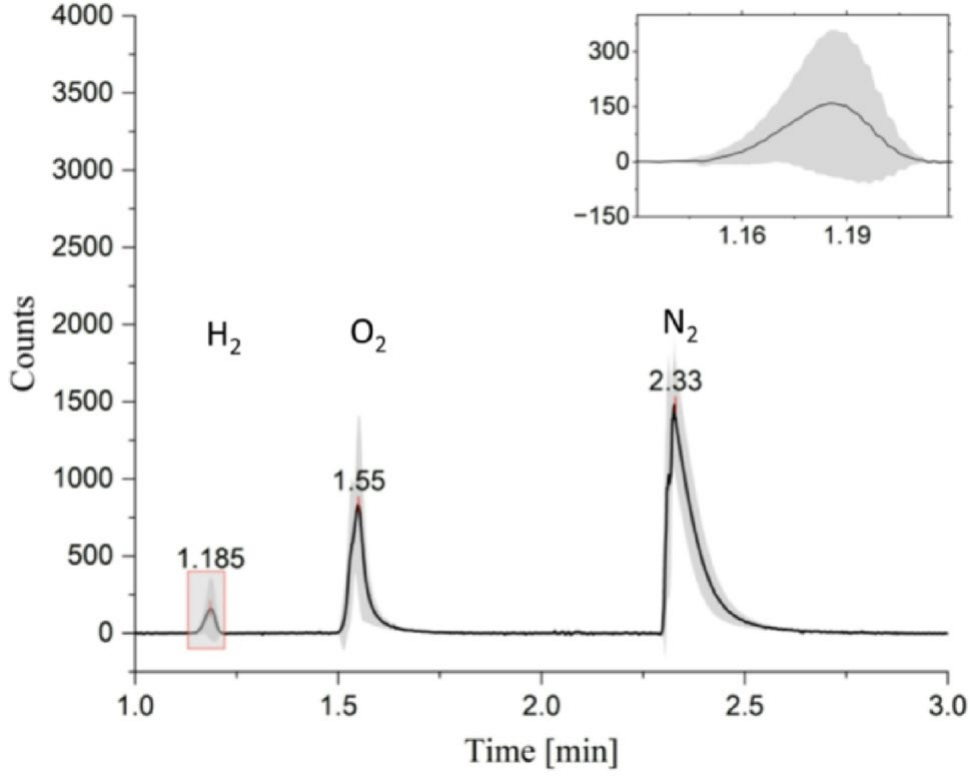
Gas chromatograph profile, example for measurements on P120_HOX in darkness with DCMU. The profiles of 12 measurements were averaged, the gray area shows the standard deviation. The peaks at 1.185, 1.55 and 2.33 min represent H_2_, O_2_ and N_2,_ respectively

After incubation, 250 µl of the gas volume from the headspace vials was drawn with a gastight syringe (Hamilton Bonaduz AG) and injected into a Hewlett-Packard HP 5890 Series II Gas Chromatograph with a thermal conductivity detector for analysis. The carrier gas was Argon (10 bar constant flow) and a 30 m·320 μm molecular sieve served as a column. The flow rate was 4.5 ml min^-1^. Inlet and detector temperatures were set to 120 ° and 150 °, respectively, the column was kept at 25°. An example for a gas chromatograph profile is presented in Fig. 2. Analytes were evaluated based on their retention time (Fig. 2).

### Chlorophyll measurements

For chlorophyll (Chl) determination, *P. lacuna* cultures were homogenized using an ultrasonic processor (UP100H, Hielscher Ultrasonics GmbH) at 100% amplitude for 1 min. One ml of culture solution was centrifuged at 4,000 × g, 4 °C for 5 min. The pellet was suspended in 1 ml methanol and incubated in darkness at 4 °C for 20–30 min. Samples were then centrifuged at 15,000 × g, 4 °C for 15 min. The UV-vis spectrum of the supernatant was recorded using a Jasco V-750 UV-Visible spectrophotometer, and Chlα concentration was calculated according to (Ritchie 2006): Chl [µg/ml] = 12.9447×(A_665_−A_720_). Note that *P. lacuna* has no other chlorophyll than chlorophyll a, like most cyanobacterial species.

### Fluorescence microscopy and quantification

After cultivation in white light to an OD_750 nm_ of 0.8 in medium with antibiotics (except wild type), the filaments were brought on a microscope slide and covered with a coverslip. Transmission and fluorescence were observed with a fluorescence microscope (ApoTome 2, Zeiss) with a 40x or 60x objective. Fluorescence recordings were made with the mCherryRed channel (red) and eGFP channel (yellow). The exposure times were manually adjusted to 15 ms for transmission, 100 ms for mCherryRed and 500 ms for eGFP. In this way, fluorescence did not reach saturation. The pixel intensities of nine measurements were estimated using ImageJ.

### Ethanol measurements

*P. lacuna* cultures were grown for 7 days at 25 °C in white light of 35 µmol m^-2^ s^-1^, brought to an OD_750 nm_ of 0.7 and treated with an Ultraturrax for 3 min at 10,000 rpm. One ml of this sample was used to measure ethanol in the medium, the other ml was extracted with a French Pressure Cell (20000 psi). This sample was centrifuged for 15 min at 9000 x g and the supernatant stored at 4 °C for less than 4 h. The reaction buffer for the enzymatic test contained 22 mM glycine, 7 mM semicarbazide and 2.5 mg/ml NAD, the pH was adjusted with NaOH to 8.6. 800 µl reaction buffer were mixed with 150 µl of extract and placed into the photometer (Jasco V-750). The absorbance at 340 nm, corresponding to NADH, was recorded continuously. After approximately 1 min, 50 µl of alcohol dehydrogenase (Merck, Darmstadt; concentration 1 mg/ml) were added. The absorbance at 340 nm, measured 2 min after this addition, was used as a readout for NADH.

### Alphafold modeling

The PsaA, PsaB, PsaC, PsaE, PsaF, PsaI, PsaJ, PsaK1, PsaK2, PsaL and PsaM *P. lacuna* sequences, the sequence of the PsaD120-HoxY and the sequence of HoxY (Hox sequences from *Microcystis aeruginosa*) were entered into Alphafold 3 (AF) (https://alphafoldserver.com/, date August 2025). The sequences are given in the supplement. The RMSD value for differences between the *P. lacuna* PSI model and the crystal structure of *Synechococcus elongatus*, PDB-code 1JB0, was 0.4002 Å. The RMSD values for *M. aeruginosa* HoxY / HoxH models compared with hydrogenase crystal structure (PDB-code 5XF9) were 0.603 Å and 0.765 Å.

## Results and discussion

### Cloning of the *P. lacuna* transformant P120_HOX containing the fusion protein PsaD-HoxY-HoxH

Solar hydrogen production has been demonstrated in the unicellular cyanobacterium *Synechocystis* sp. PCC 6803 (Appel et al. 2020). In that study, the *hoxY* and *hoxH* genes from *Synechocystis* were cloned to generate a fusion protein in which HoxY was linked to Glu106 of the photosystem I subunit PsaD, replacing the 35 C-terminal amino acids of PsaD. HoxY and HoxH are the core proteins of the NiFe hydrogenase and are apparently sufficient for H_2_ production or H_2_ degradation. In the light and with DCMU, the transformant produced up to ten times more H_2_ than the wild type. We constructed *P. lacuna* transformants in which PsaD is linked to HoxY from either *Microcystis aeruginosa*, *P. lacuna*, *Synechocystis*, *Oscillatoria lacuna* (Sinetova et al. 2025) or *Cupriavidus necator*. In all transformants the *hoxY* and *hoxH* coding regions are located within the *psaD* locus and HoxY is covalently linked to position 120 of *P. lacuna* PsaD. This position is equivalent to Glu106 of *Synechocystis* PsaD. Full integration into *P. lacuna* by homologous recombination and complete segregation was demonstrated by PCR using outer and inner primers. Our present study concentrates on hydrogen measurements of wild type and the transformant with *M. aeruginosa* HoxY/HoxH, the first transformant for which hydrogen production was established. This transformant is termed P120_HOX. The gene arrangement is given in Fig. 1 and cloning details are given in the supplement.

### Oxygen and chlorophyll measurements

**Fig. 3:**
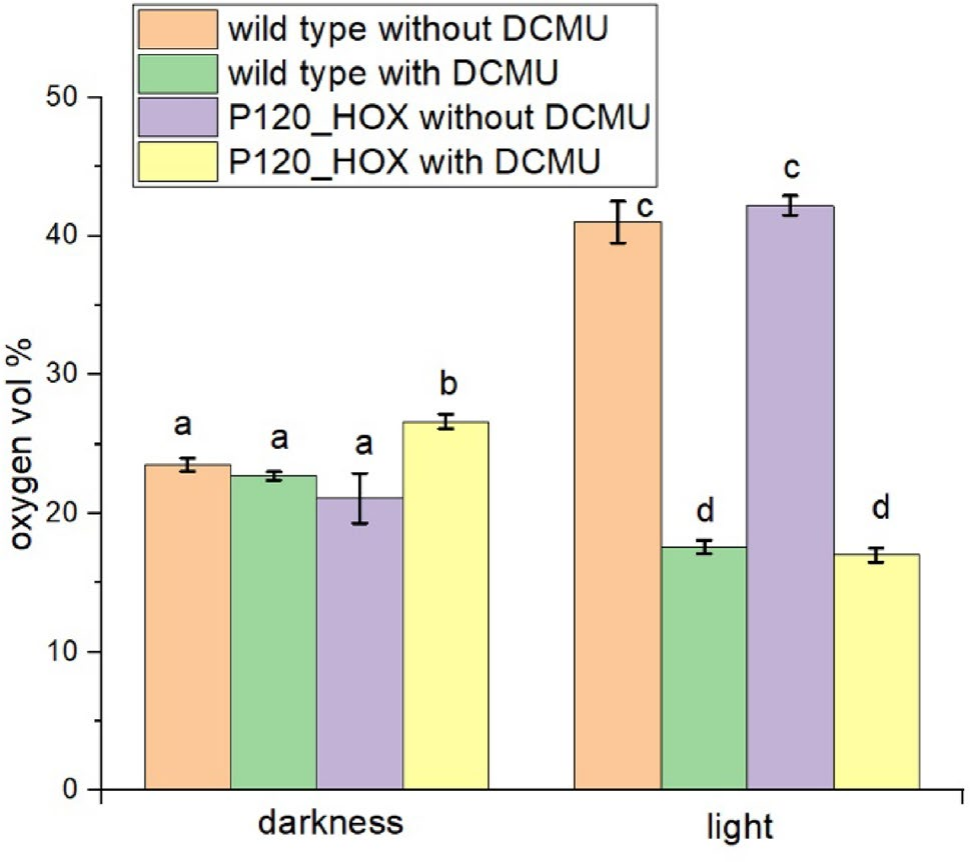
Oxygen in vol % as measured in the headspace by the GC. Medium without filaments (i.e. air) had 23.6 ± 1 vol %. DCMU concentrations were 100 µM. Mean values ± SE of 11 measurements. Significance was tested pairwise by one sided t-tests (*p* < 0.05). Identical letters indicate that differences are not significant, different letters indicate that differences are significant

For comparative gas measurements of wild type and P120_HOX in light and darkness, headspace vials were used in which the filaments settle to the ground. The gas chromatograph provides values for H_2_, O_2_ and N_2_ in the headspace (Fig. 2). We first present the O_2_ values that reflect the effects of photosynthesis and which give an estimate for O_2_ in the solution under different conditions (Fig. 3). The O_2_ levels in all dark samples were comparable with values as measured from air (in the same vials with medium) which were 23.6% ± 0.5%. Since we used no oxygen removing chemical, these results were in the expected range. We also found no significant differences between the wild type without DCMU (23.5 ± 0.2) and the other samples with the exception of P120_HOX with DCMU, which has a slightly increased mean value of 26.5 ± 0.5. We therefore expect that all dark samples contain about equal oxygen concentrations in the medium. In both samples kept in the light without DCMU, oxygen levels rose to high values above 40%, indicating significant photosynthesis activity. Light plus DCMU (which blocks PSII) resulted in a reduction of oxygen to below the air values. Photosynthetic O_2_ production must be completely blocked by DCMU and the decrease to below dark levels is explained by elevated respiration (because of induced stress in the light), result of the Mehler reaction (Mehler 1951) or photorespiration (Zhou et al. 2021). The significant reduction of oxygen indicates that oxygen consumption by filaments can be detected in the headspace, despite the diffusion distance of around 3 cm.

**Fig. 4:**
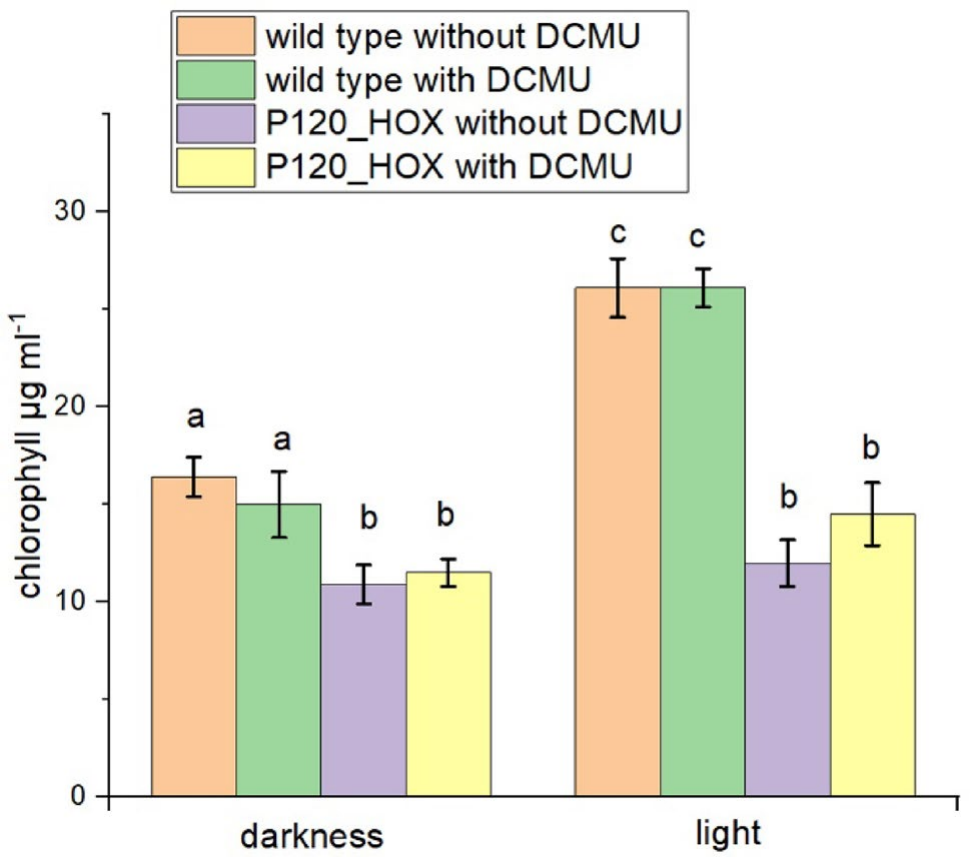
Chlorophyll in µg/ml of the same samples as in Fig. 3. Mean values ± SE of 11 measurements. Significance was tested pairwise by one sided t-tests (*p* < 0.05). Identical letters indicate that differences are not significant, different letters indicate that differences are significant

Chlorophyll measurements after the 2 d of specific incubation showed that P120_HOX had consistently lower mean chlorophyll levels than the wild type when compared under the same conditions. Wild type levels ranged from 15 to 26 µg/ml, whereas P120_HOX ranged between 11 and 15 µg/ml (Fig. 4). In both light-treated wild type samples the chlorophyll levels were roughly 1.5 fold higher than in the dark samples, the differences between dark and light were significant. Since no photosynthesis is expected with DCMU, the increased chlorophyll level in the DCMU sample indicates respiration driven chlorophyll synthesis, possibly as compensation for the lost linear electron flow. Light in the presence of DCMU did not increase chlorophyll levels as compared to the dark levels in neither strain.

### Hydrogen production in wild type and P120_HOX

**Fig. 5:**
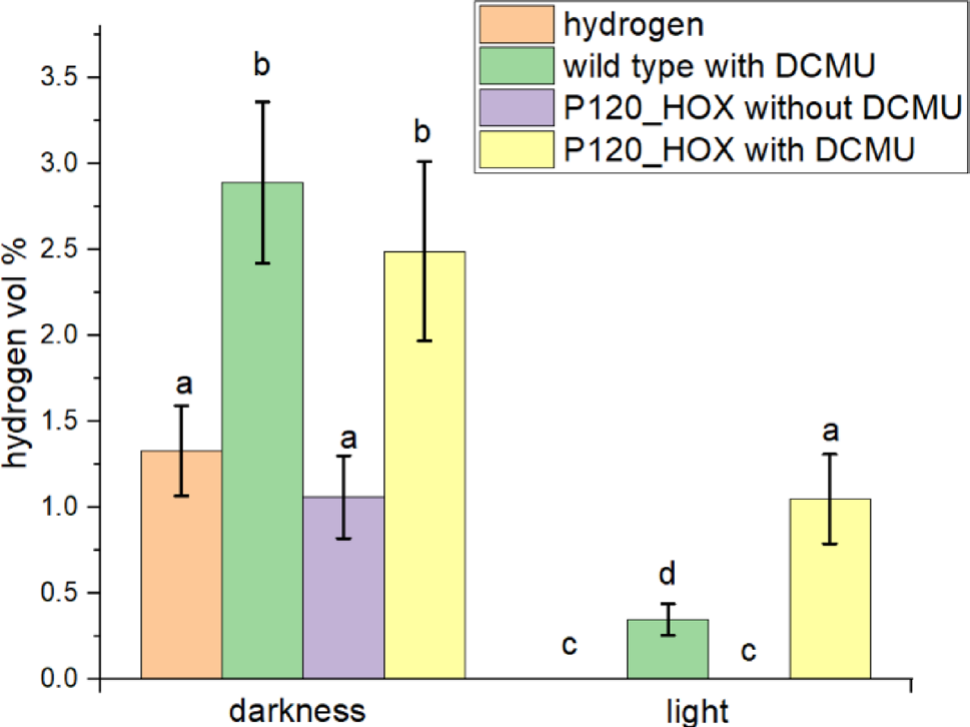
Hydrogen vol % as measured in the headspace by the GC. DCMU concentrations were 100 µM. Mean values ± SE of 11 measurements. Significance was tested pairwise by one sided t-tests (*p* < 0.05). Identical letters indicate that differences are not significant, different letters indicate that differences are significant

**Fig. 6:**
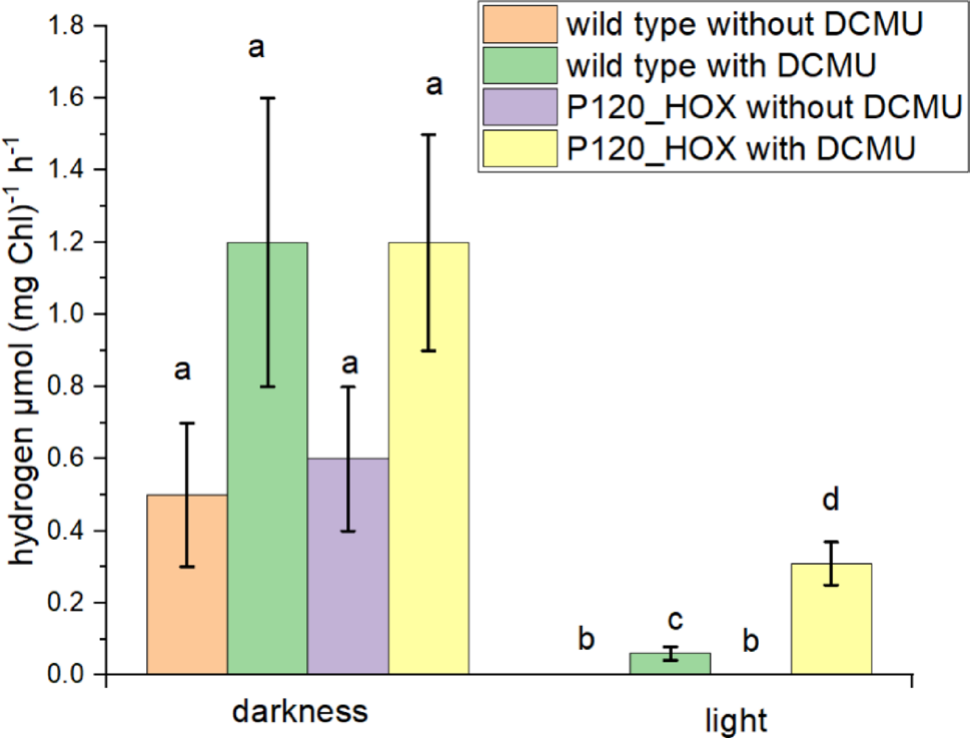
Hydrogen in µmol (mg Chl)^−1^ h^− 1^. Mean of ± SE of 11 measurements. DCMU concentrations were 100 µM. Significance was tested pairwise by one sided t-tests (*p* < 0.05). Identical letters indicate that differences are not significant, different letters indicate that differences are significant. For the estimarion of µmol hydrogen, see Methods section

Hydrogen measurements are presented in Figs. 5 in vol % and in Fig. 6 as µmol (mg Chl)^-1^ h^-1^ for comparison with published data. The genome of *P. lacuna* contains genes for Ni/Fe hydrogenase subunits HoxH and HoxY (see sequences in the supplementary document 2). In addition, there are genes for the diaphorase proteins HoxE, HoxF, and HoxU, which participate in fermentative H_2_ synthesis (Eckert et al. 2012; Gutekunst et al. 2018; Wang et al. 2023). We indeed found H_2_ production in dark grown wild type *P. lacuna* (Figs. 5 and 6) of about 0.5–1.2 µmol (mg Chl)^-1^ h^-1^. This is higher than the 0.1 µmol (mg Chl)^-1^ h^-1^ reported for *Synechocystis* (Appel et al. 2020) and within the range of reported values of other cyanobacteria without heterocysts (Dutta et al. 2005). The large error bars indicate large variations between different hydrogen measurements which can be due to variations in hydrogen losses during cultivation or sample transfer. A difference between wild type and transformant was not obvious. The two-fold increases of the mean values by DCMU were insignificant. Since we used no O_2_ removing chemical such as argon, we must assume that O_2_ is dissolved in the medium and that the hydrogenase is independent of the presence of O_2_, (note that normal O_2_ levels were detected in the headspace). This stands, however, in contrast to the general assumption that cyanobacterial hydrogenases are O_2_ sensitive in vitro. We assume that the intracellular O_2_ concentrations are reduced by e.g. the cell wall or that the hydrogenase is somehow shielded from external O_2_.

In the illuminated samples without DCMU we detected no H_2_, neither in the wild type nor in P120_HOX. We assume that the O_2_ as produced during photosynthesis inhibits internal and recombinant hydrogenases, due to its high concentration in the cell. Light in the presence of DCMU (inhibits PSII) gave values of 0.06 µmol (mg Chl)^-1^ h^-1^ for the wild type and 0.31 µmol (mg Chl)^-1^ h^-1^ for P120_HOX, the differences were significant. Photohydrogen production in the *Synechocystis* system were reported to be 0.79 µmol (mg Chl)^-1^ h^-1^ (Appel et al. 2020). Our results are interpreted as indication for photohydrogen production by the overexpressed HoxY/HoxY hydrogenase subunits, although the 5 fold increase in H_2_ levels of P120_HOX are still below the dark levels of wild type and P120_HOX and clearly too low for applications in solar hydrogen production. We conclude that *P. lacuna* can be used as a model system for photohydrogen production in a filamentous cyanobacterium and we predict that further optimization can be tested in this model organism.

### Modeling shows that the minimal distance between FeS clusters is around 29 Å

**Fig. 7:**
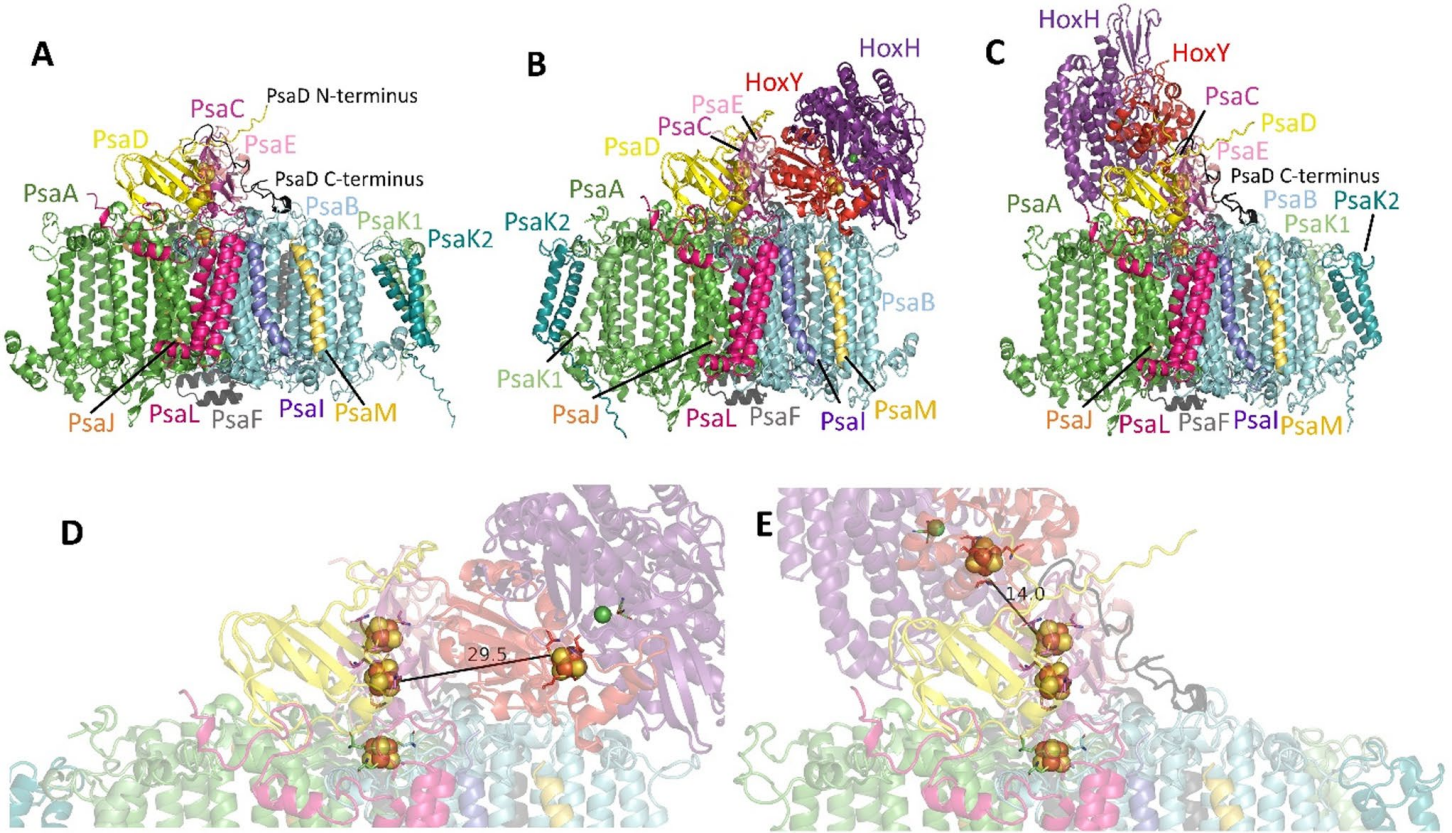
Alphafold models of *P. lacuna* PSI (**A**), P120_HOX (**B**,** D**) and P120_HOX_P121-155 (**C**,** E**). All subunits are drawn in different colors. FeS clusters and the active Ni atom of HoxH are from alignments with 1JB0 and 5XF9, other cofactors such as chlorophylls are omitted. Note the different orientation of HoxY/H vs. PSI in (**B**,** C**) and the different position of PsaK1/PsaK2 in (**A**, **B**). Distances between closest FeS clusters of PSI and HoxY are shown in (**D**,** E**)

One reason for the still low photohydrogen production in the present and previous study could be the rather large distance between FeS clusters of PSI and HoxY that has already been discussed before (Appel et al. 2020). With Alphafold (Jumper et al. 2021) it is possible to generate models with good prediction rates. We used Alphafold 3 (https://alphafoldserver.com) to test for proposed FeS distances of our *P. lacuna* system. In initial studies, models were built using PSI subunits of *P. lacuna* alone. The *P. lacuna* PSI model (Fig. 7A) matched very well with the crystal structure of *Synechococcus elongatus* PSI (1JB0) with an RMSD of 0.382 Å for the protein backbones. The Cys residues coordinating the FeS clusters F_X_, F_A_ and F_B_ were located at the same positions. *P. lacuna* has an additional subunit PsaK2, which was positioned at the periphery of PSI next to PsaK1 (see also Fig. 7A).

A model based on the HoxY and HoxH sequences of *M. aeruginosa* was used for comparison with the crystal structure of *Hydrogenophilus thermoluteolus* hydrogenase (PDB: 5XF9). Here, an RMSD of *R* = 0.565 Å was obtained. We therefore assume that a model for the P120_HOX protein complex is a good prediction to estimate FeS-FeS distances.

In the P120_HOX model, HoxY extended to the lumenal side with HoxH bound to HoxY. PsaK1 and PsaK2 were apparently displaced from their original position and located on the opposite side of PSI. Of the three FeS clusters of PSI, F_A_ was closest to the FeS cluster of HoxY, the edge to edge distance was 29.5 Å (Fig. 7B and D). The ranked models differed by the orientation of HoxY/H versus PSI by motions around the PsaD-HoxH linker region. The distances in all 5 ranked models ranged from 29.5 Å to 45.5 Å (Supplementary Table 1). These distances are within the range discussed by Appel et al. for their system (Appel et al. 2020; Wang et al. 2023). A distance of 29.5 Å would be too large for efficient electron transfer. We assume that movement of the HoxY/H complex around PSI could reduce the FeS–FeS distance further for short time, which would explain why photohydrogen production occured at all.

After the Alphafold system was already set up we asked whether models for lower distances could be generated. Our first trials did however not lead to an improvement of the relevant distances: We generated 24 models in which HoxY was covalently attached to either the N- or C-termini of PSI proteins. In 12 models, we attempted to integrate HoxY into PSI proteins at selected positions. In all models, distances were either above 35 Å or protein folding was severely affected.

By the end of our Alphafold session one more model was generated by accident in which the C-terminal 35 amino acids of PsaD were fused to the C-terminus of HoxY in P120_HOX. This model is termed P120_HOX_P121-155 here (Fig. 7C and E). To our initial surprise, the closest edge-to-edge distances between FeS clusters of PSI and HoxY were in the range of 14 Å. In the ranked models, similar distances were found. The C-terminal 35 amino acids of PsaD in P120_HOX_P121-155 occupied nearly the same position next to PsaB as in the wild type protein, despite the insertion of HoxY. In this way, HoxY was drawn into a completely different position as compared to P120_HOX. In addition, PsaK1 and PsaK2, were located at the position they have in the wild type model. The score values that predict the confidences of the models were 0.70 to 0.73 for ipTM and 0.74 to 0.77 for pTM. These values indicate good confidence for the overall fold and thus reliability of the models.

A distance of around 14 Å is typical for FeS–FeS electron transfer in respiratory chains (Martin et al. 2017) and in PSI (Jordan et al. 2001). It is therefore very likely that highly efficient electron transfer takes place in P120_HOX_P121-155, which could lead to elevated H₂ production.

We believe that in biohydrogen research, protein modeling can make a significant contribution to future improvements.

### sfGFP expression demonstrating strong promoter activity

To gain a better understanding of the psaD locus and expression under the *psaD* promoter, we used sfGFP to visualize different constructs. One question was whether PsaD fusion proteins (such as the PsaD-HoxY described above) would be expressed at high levels. Another question was whether and to what extent a gene located 3′ of psaD could be driven by the promoter.

**Fig. 8:**
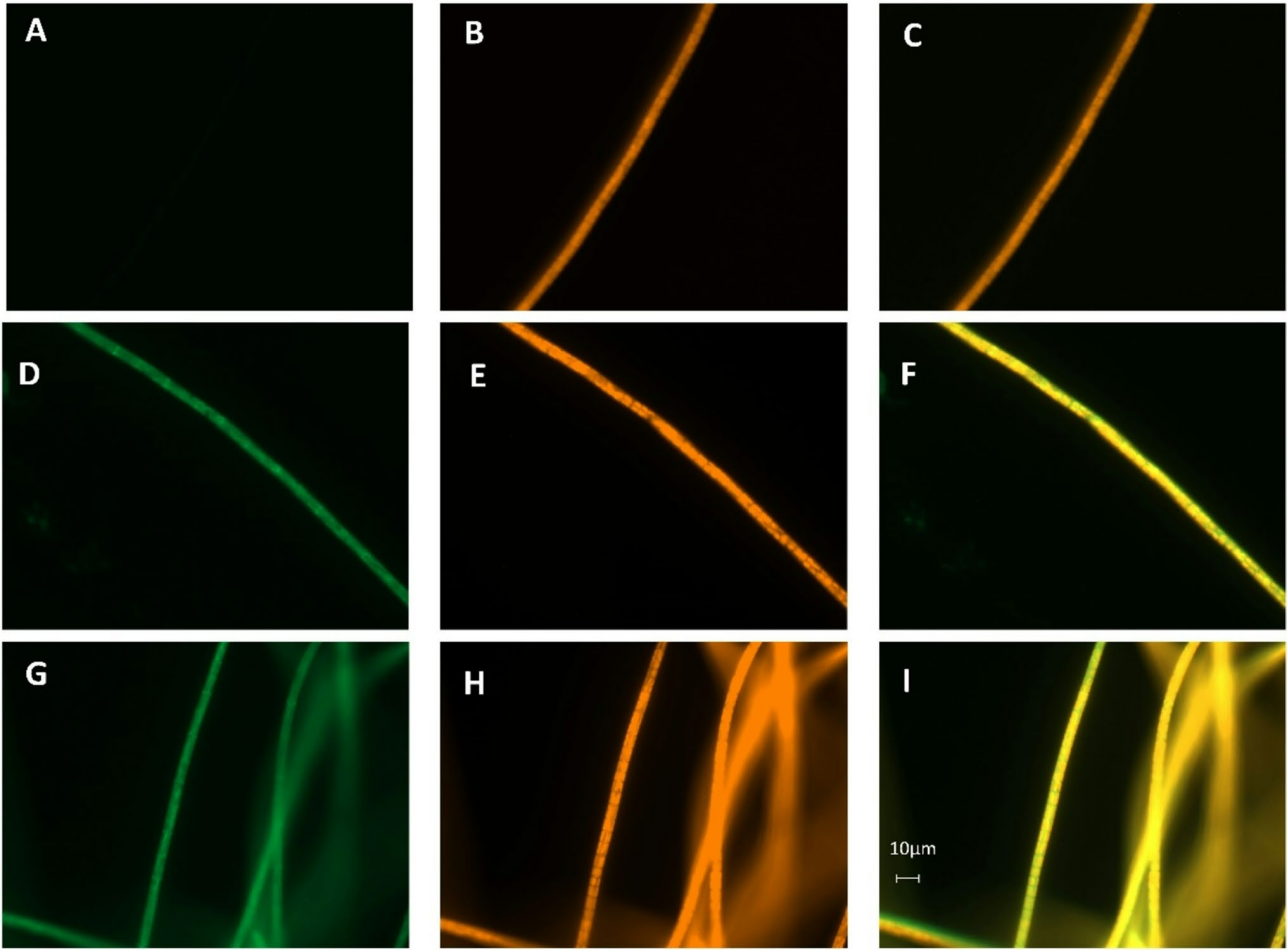
Yellow (**A**,** D**,** G**) and red (**B**,** E**,** H**) fluorescence of wild type (**A–C**), P155_GFP (**D–F**) and P155_SD_GFP, recorded with 60x objective

We generated one construct in which sfGFP is covalently linked to the C-terminus of PsaD, and another construct containing a stop codon and a Shine–Dalgarno sequence between the two genes. These constructs are termed P155_GFP and P155_SD_GFP, respectively. Fluorescence images are shown in Fig. 8. In all three samples (wild type, P155_GFP, and P155_SD_GFP), two regions of high chlorophyll density were observed across the cross section of a cell. The sfGFP signal matched the chlorophyll fluorescence only partially in both P155_GFP and P155_SD_GFP. In both cases, regions with yellow fluorescence only were observed, as seen most clearly in the merged images. This fluorescence cannot be due to background fluorescence, as it is absent in the wild type. Since in P155_GFP sfGFP is covalently bound to PsaD, we conclude that not all PsaD proteins are integrated into PSI, at least not in the form of the sfGFP fusion protein.

We quantified the fluorescence signals and compared these with previously published sfGFP lines of our group (Weber et al. 2022). These quantified signals are given in Table 1. The highest fluorescence level was obtained with sfGFP under control of the endogenous *cpcB* promoter. The super *cpc560* promoter (from *Synechocystis* sp. PCC 6803) yielded the lowest sfGFP signal in the present study, despite the high expression levels reported in other cyanobacteria (Zhou et al. 2014). The fluorescence values for P155_SD_GFP and P155_GFP were intermediate between those obtained with the *cpc560* and *cpcB* promoters (Weber et al. 2022). Expression of sfGFP as a separate protein from PsaD resulted in stronger fluorescence than the PsaD–sfGFP fusion.

The *psaD* promoter currently shows the most suitable characteristics for expression in *P. lacuna*, although expression levels are lower than those obtained with the cpcB promoter, for which segregation times are very long.

**Table 1:** Fluorescence intensities of wild type and sfGFP expressing transformants

Wild type	P155_SD_GFP	P155_GFP	MH1	AK1
5.2 ± 0.2	24 ± 2	17 ± 2	40 ± 3	12 ± 0.4

Fluorescence images were taken with a 40 x objective. MH1: transformant in which the sfGFP is driven by the endogenous cpcB promoter (Weber et al. 2022); AK1: the sfGFP driven by the cpc560 promoter (Zhou et al. 2014), in both cases cloned into a neutral site (Weber et al. 2022). Mean ± SE, of 9 measurements normalized to a maximum pixel intensity of 100. Each sample is significantly different to every other sample (one sided t-test, *p* < 0.05)

### Ethanol production

During earlier trials to establish *P. lacuna* for ethanol production, we expressed pyruvate decarboxylase (PDC) and alcohol dehydrogenase (ADH) under the control of the promoter of the kanamycin resistance cassette, because *P. lacuna* strains transformed with this cassette exhibit high levels of kanamycin resistance. However, ethanol in this transformant was not detectable above wild type level (Nies 2019). Here, we placed both genes under the control of the *psaD* promoter by positioning the coding sequences 3′ of *psaD*. The resulting transformant is termed P155_PDC_ADH (P155 stands for full length PsaD with 155 amino acids).

**Fig. 9:**
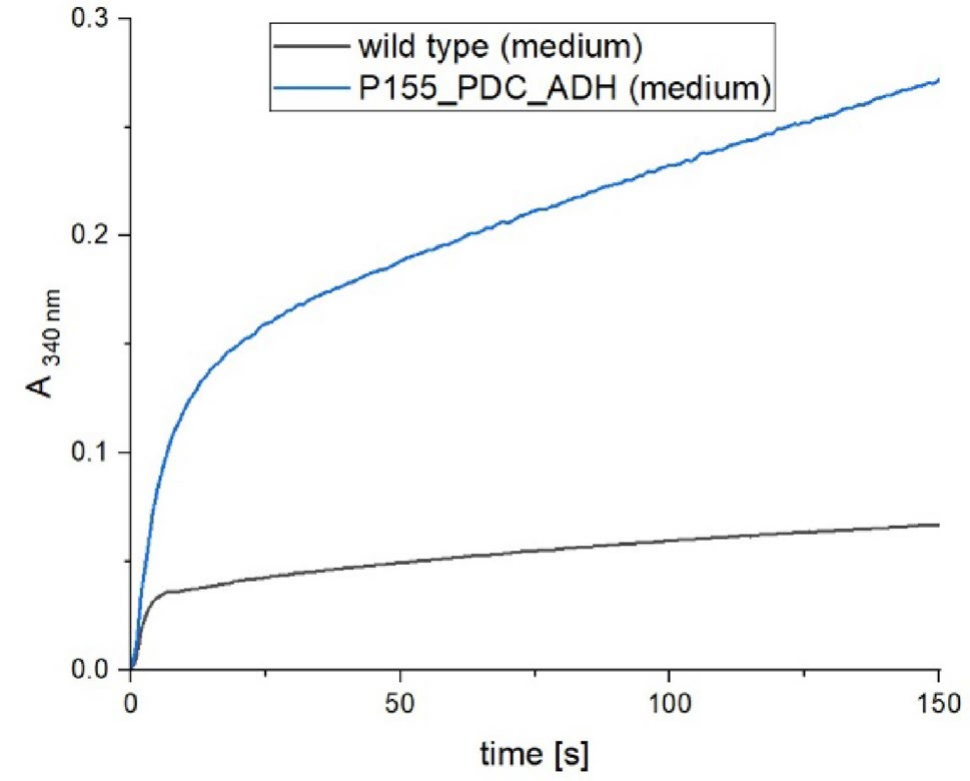
Ethanol measurement, A _340 nm_ time course for NADH increase after addition of alcohol dehydrogenase

Ethanol was quantified using a conventional enzyme assay in which NADH production was monitored as an increase in A340 over 2 min after addition of commercial ADH (see example in Fig. 9). The absorbance values were converted into ‰ (v/v) concentrations using an equilibration measurement with 0.01‰ (v/v) ethanol. For each sample, measurements were taken from both the growth medium and the cell extract (obtained by French press).

For both medium and extract, we obtained a small but significant signal in wild-type filaments. Such signals were consistently observed in other measurements performed in the laboratory. Since the *P. lacuna* genome lacks ADH and PDC, we assume that the signal may correspond either to another alcohol or to ethanol produced via alternative pathways.

The values obtained for the transformant were both 0.1‰, significantly higher than the wild-type values. The combined values for growth medium and extract (Table 2) correspond to 3.4 mM ethanol, which is much higher than the 500 nM reported in the first study on ethanol production in cyanobacteria (Deng et al. 1999), but lower than the approximately 5 mM reported in a later study (Dexter et al. 2009).

**Table 2:** Ethanol measurements in wild type and P155_PDC_ADH

	Wild type	P155_PDC_ADH medium
Medium	0.017 ± 0.003‰ (v/v)	0.10 ± 0.02‰ (v/v)
Extract	0.016 ± 0.007‰ (v/v)	0.10 ± 0.01‰ (v/v)

Mean values of 3 samples ± SE. Values of P155_ADH_PDC and wild type are significantly different (one-sided t-test, *p* < 0.01)

Our study shows that ethanol production is feasible in *P. lacuna* by bringing the relevant genes under the control of the *psaD* promoter. Biofilm formation could increase the cell densities and thereby lead to a higher production of ethanol and could allow easy separation of medium and filaments.

## Conclusions

Our present study contributes to the development of filamentous cyanobacteria as hosts for genetic engineering aimed at improving biohydrogen and bioethanol production. In this context, our results confirm previous findings on photohydrogen and bioethanol production obtained with unicellular cyanobacterial species.

One advantage of filamentous species is their pronounced ability to form biofilms compared with unicellular species (Bozan et al. 2022). *P. lacuna* forms biofilms on different surfaces and under various environmental conditions. Bioreactors based on biofilm systems could enable continuous operation and facilitate separation of the growth medium from the cyanobacterial biomass, for example allowing ethanol-containing medium to be removed while retaining the cells. In addition, improved photosynthetic efficiency could allow biomass production to be combined with biofuel production.

The efficient commercial use of cyanobacteria for biofuel production will require substantially higher production rates, which could be achieved through further genetic engineering. The upper limit of ethanol production may be constrained by the ethanol tolerance of the cyanobacterium; however, this limitation could potentially be overcome by continuous separation of the medium from the cell mass.

One possible strategy for improving photohydrogen production is outlined in this study. Further basic research could increase hydrogenase efficiency, and structural modeling may help to address limitations in electron transfer distances.

The establishment of recombinant filamentous cyanobacteria for biohydrogen and bioethanol production is supported by the natural competence of *P. lacuna* and the use of the strong *psaD* promoter demonstrated in this work. Despite the need for further improvements at the molecular level, including higher production yields and better growth under natural conditions, the design of bioreactors suitable for filamentous species will also need to be optimized.

One option could be to transfer the knowledge obtained with filamentous systems such as *P. lacuna* to *Arthrospira* (Abreu et al. 2023). Alternatively, the biofilm-forming capacity of *P. lacuna* itself could be exploited, enabling high cellular densities and facilitating separation of the filaments from the surrounding medium.

## Supplementary Information

Below is the link to the electronic supplementary material.

Supplementary Material 1

Supplementary Material 2

Supplementary Material 3

## Data Availability

The datasets generated during and/or analyzed during the current study are available from the corresponding author on reasonable request.
