# Supplementary material for: Increased hydrogen and ethanol production in transformants of the filamentous cyanobacterium Phormidium lacuna
Source: Arch Microbiol. 2026 May 30;208(8):414. doi: 10.1007/s00203-026-04932-4 (PMC13222304; doi:10.1007/s00203-026-04932-4)
Supplement: Supplementary file 1 — Supplementary Material 1 [file 203_2026_4932_MOESM1_ESM.docx]

**Supplementary Document 1**

**Cloning of Transformation vectors**

All transformation vectors were constructed for insertion into the *P. lacuna* genome by homologous recombination. All vectors contain the flanking regions of psaD in the genome and all besides pGEMTeasy_psaD2k and pGEMTeasy_psaD_SD_PDC-ADH_GmR contain a kanamycin resistance cassette. Cloning was performed using standard molecular techniques (Sambrook et al.) (Sambrook et al. 2001) or cited methods. An overview of the genetic situation in the transformants after integration into the genome of *P. lacuna* is presented in Figure 1. After transformation and segregation, integration of the insert between the homologous sequences into the genome of *P. lacuna* by homologous recombination and completeness of segregation was confirmed using inner primers (GGTTTCAGACAGTCTTCG (fwd) and CAAGGAGAGTACCTTGAG (rev)) and outer primers (CCGCTCTCGTTACAAGAATG (fwd) and GACACTCTCGCAGAATTGC (rev)) for all vectors.

**Vector pGEMTeasy_psaD2k**

This vector contains the psaD coding sequence and 1000 bp upstream and 1000 bp downstream sequences for later homologous integration into the *P. lacuna* genome. The 2 kDa sequence was amplified from *Phormidium lacuna* HE10DO using primers GAGTTGGTGGTGGATATTC (fwd) and GAGCATCGTATCTTCGCTG (rev) by colony PCR and cloned into pGEMTeasy (Promega) by TA cloning. The homologous sequences range into a histidine kinase gene at the 5' end and a gene for an anthranilate synthase subunit at the 3' end.

**Vector pGEMTeasy_psaD2k-KanR**

This vector contains an additional Kanamycin resistance cassette. pGEMTeasy_psaD2k was linearized using primers AGTCACCACCTGCAGTCAATTAGCGGTGTAGGGTTGTTTG (fwd) and TGACTAGCTAGCATCGGTTCCGCTTGTCAC (rev). This PCR eliminates the psaD stop codon. The KanR cassette was amplified with primers AGTCACGCTAGCTTTGCTTTGCCACGGAACG (fwd) and AGTCACCACCTGCAGATAATTCCCCGGATCCGTCGA (rev) and the two sequences ligated by sticky end cloning with restriction enzymes PaqCI and BmtI. The Kan resistance cassette is positioned 3' of the psaD coding sequence.

**Vector pGEMTeasy_psaD2k-Glu120-hoxYH-PCC9806**

This vector encodes for a PsaD-HoxY/HoxH fusion, where the N-terminus of *Microcystis aeruginosa* HoxY is fused to psaD at the position of Glu 120. HoxH is encoded 3' of HoxY, separated by a Shine Dalgarno sequence. The hydrogenase gene hoxY of *Microcystis aeruginosa* (from the Pasteur stock collection) was amplified by colony PCR using the primers ACGACGGTTTTAGTCATGAATTAATCTCCTTTAACCAAATTTAATCATTTCTCGTCCTG (fwd) and CTCCATCCCAAAGATGGTGTGTTCCCCGAGATGTCTAAAATTCGCTTCGCTAC (rev)

The *Microcystis aeruginosa* hoxY gene was amplified using the primers TCAGAGATTTTGAGACACAACGTGGCTTTCTTAACCCCTTTGTCGAGTATTGATCACCTC (fwd) and GCAGGACGAGAAATGATTAAATTTGGTTAAAGGAGATTAATTCATGACTAAAACCGTCGTTATCGATCCC (rev). The vector pGEMTeasy_psaD-KanR was amplified using the primers

GAAAGCCACGTTGTGTCTC (fwd) and CTCGGGGAACACACCATCTTTG (rev), thereby reducing the psaD coding region to position Glu120. The three constructs were fused together by Gibson assembly (Gibson et al. 2009). The vector is abbreviated by pNS18, the transformant is termed plNS18 or P120_HOX (for *P. lacuna* PsaD position 120 - HoxY - HoxH).

**Vector pGEMTeasy_psaD2k-SD-sfGFP**

This vector is designed for sfGFP (Pédelacq et al. 2006) expression under the control of the psaD promoter. The sfGFP coding region is 3' of psaD, separated by a Shine Dalgarno sequence. The vector pGEMTeasy_psaD2k-KanR was linearized using primers AGCGGTGTAGGGTTGTTTG (fwd) and GGTACCaattccccggatc (rev). The sfGFP sequence was obtained from synthetic puc19cpcB_sfGFP, a vector for transformation of *P. lacuna* published earlier (Weber et al.) (Weber et al. 2022) using the primers GTTCAGCGGCAAACAACCCTACACCGCTTAAAGGAGATTAATTCATGAGCAAAGG (fwd) and GGTCGACGGATCCGGGGAATTGGTACCTTATTTGTAGAGCTCATCC (rev). Both sequences were fused and circularized by Gibson assembly (Gibson et al.) (Gibson et al. 2009). The transformant is termed P155_SD_GFP (for full length PsaD - Shine Dalgarno - sfGFP).

**Vector pGEMTeasy_psaD2k-dSTOP-sfGFP**

This vector expresses for a psaD - sfGFP fusion protein. Primers AGCGGTGTAGGGTTGTTTG (fwd) and ATGAGCAAAGGAGAAGAACTTTTCAC (rev) were used to amplify the pGEMTeasy_psaD2k-SD-sfGFP. The product was recircularized. In this way, the stop codon and the Shine Dalgarno between the psaD sequence and the sfGFP coding regions were removed. The transformant is termed P155_GFP (for full length PsaD - sfGFP).

**Vector pGEMTeasy_psaD_SD_PDC-ADH_GmR**

This vector was designed for ethanol production in *P. lacuna.* It contains a gentamycin resistance cassette (GmR) (McBride et al.) (McBride et al. 1990). The pyruvate decarboxylase (PDC) gene from *Zymomonas mobilis* (Boismier et al.) (Boismier et al. 2025) was amplified using primers CAACAAGAAGACTCGACCTAGAGGAGCTTGTTAACAGGC (fwd) and CAACAAGAAGACTGTTATGAGTTATACTGTCGGTACCTATTTAGCG (rev). The alcohol dehydrogenase (ADH) gene from *Z. mobilis* was amplified using the primers CAACAAGAAGACACTTAGAAAGCGCTCAGGAAGAGTTC (fwd) and CAACAAGAAGACCTCTAGGTCGACTGAGGTTATAGCTATGGC (rev). Both were cloned next to each other into a pGEM (Promega) cloning vector (5' PDC and 3' ADH) by Golden Gate cloning (Engler et al.) (Engler et al. 2008). The PDC-ADH construct (both genes) was amplified using TACACCGCTTAAAGGAGATTAATTCATGAGTTATACTGTCGGTACCTATTTAGCG (fwd) and GAACAGGCTTATGTCAAGATGTCTAGCAGGTCGACTTAGAAAGCG (rev) and the Gentamycin resistance cassette (GmR) was amplified using GGAAGACAACTTAATGTCCGTGACTTAGGTGGCGGTACTTGG (fwd) GCTTTCTAAGTCGACCTGCTAGACATCTTGACATAAGCCTGT (rev). PDC/ADH and GmR were cloned by Gibso assembly (Gibson et al.) (Gibson et al. 2009) into pGEMTeasy_psaD. In the construct, GmR is 3' of ADH and 5' of the homologous region. A Shine Dalgarno sequence was introduced 5' of the PDC gene by amplification with GTCACGGACATTAAGTTGTC (fwd) and GAATTAATCTCCTTTAAGCGGTGTAGGGTTGTTTG (rev) and recirularization. The transformant is termed P155_PDC_ADH.

**References**

Boismier, EC, Aboulnaga, EA, & TerAvest, MA (2025). Zymomonas mobilis: bringing an ancient human tool into the genomic era. *Current Opinion in Biotechnology, 92*, 103257, doi:<https://doi.org/10.1016/j.copbio.2025.103257>.

Engler, C, Kandzia, R, & Marillonnet, S (2008). A one pot, one step, precision cloning method with high throughput capability. *PLoS One, 3*(11), e3647, doi:10.1371/journal.pone.0003647.

Gibson, DG, Young, L, Chuang, R-Y, Venter, JC, Hutchison, CA, & Smith, HO (2009). Enzymatic assembly of DNA molecules up to several hundred kilobases. *Nature Methods, 6*(5), 343-345, doi:10.1038/nmeth.1318.

McBride, KE, & Summerfelt, KR (1990). IMPROVED BINARY VECTORS FOR AGROBACTERIUM-MEDIATED PLANT TRANSFORMATION. *Plant Mol.Biol., 14*(2), 269-276, doi:10.1007/bf00018567.

Sambrook, J, & Russell, DW (2001). *Molecular Cloning. A Laboratory Manual. 3rd edition.*: Cold Spring Harbor Laboratory Press.

Weber, N, Hofmeister, M, Wunsch, N, Kohler, A, Kaster, AK, Vollmers, J, Kachel, B, Mack, M, & Lamparter, T (2022). Natural Transformation, Protein Expression, and Cryoconservation of the Filamentous Cyanobacterium Phormidium lacuna. *J Vis Exp*(180), doi:10.3791/63470.
