# Supplementary material for: Increased hydrogen and ethanol production in transformants of the filamentous cyanobacterium Phormidium lacuna
Source: Arch Microbiol. 2026 May 30;208(8):414. doi: 10.1007/s00203-026-04932-4 (PMC13222304; doi:10.1007/s00203-026-04932-4)
Supplement: Supplementary file 2 — Supplementary Material 2 [file 203_2026_4932_MOESM2_ESM.docx]

Supplementary Table 1. Distances between FeS clusters of PS I and HoxY in structural AF3 ranked models. The distances are estimated between closest sulfur residues of Cys residues of F_A_ or F_B_ and of HoxY and are comparable with the edge to edge distances between FeS.

|  | P120_HOX | P120_HOX_P121-155 |
| --- | --- | --- |
| model0 | 29.5 Å | 14.0 Å |
| model1 | 30.7 Å | 14.6 Å |
| model2 | 44.9 Å | 17.2 Å |
| model3 | 45.5 Å | 12.7 Å |
| model4 | 41.4 Å | 21.8 Å |
