# Supplementary material for: Increased hydrogen and ethanol production in transformants of the filamentous cyanobacterium Phormidium lacuna
Source: Arch Microbiol. 2026 May 30;208(8):414. doi: 10.1007/s00203-026-04932-4 (PMC13222304; doi:10.1007/s00203-026-04932-4)
Supplement: Supplementary file 3 — Supplementary Material 3 [file 203_2026_4932_MOESM3_ESM.docx]

**Supplementary document 2**

**Protein and DNA sequences, FASTA formatted**

Protein sequences

>Phormidium lacuna HoxE

MTPTQTSTAAKSSQNAPKKDGKPDKRFKVLDITMKRNQYRQDALIEILHKAQEAFGFLEEDVLSYIAHKLQLPLSQVYGVATFYHLFSLKPSGAHTCVVCLGTACYVKGGGDILKAVEGDVGIAAGETTEDGQVSIVTARCIGACGIAPAVVYDGKVAGQQTPEQTCDRIHSWKQEDS

>Phormidium lacuna HoxF

MERAELLEQATAAKQAQKRIRIHCCTSTGCQASQSLDVKKQLDAAIKTHHLGDTVEAVGVGCMGFCGQGPMVEIEDTEAPAEKKHYQKVTPEQAESIIGSLNGSGEADAIQGDPNHPFFSRQLLIVREHSGRIDPERIDEYLAVGGYQALHHAIYEMSPADVVQEITQSGLRGRGGAGYPTGLKWATVAKMPPGQKYIICNADEGDPGAFMDRSVLESDPHRVLEGMAIAGYAVGATQGYIYVRAEYPLAITRLQKAIQQAKRKHCLGAQIFDSPVDFTIEIRVGAGAFVCGEETALISSVEGGRGNPRPRPPYPAVSGLHGCPTLINNVETLGNISPIIRNGAEWFAGIGTERSKGTKIFSLTGKIRNNGLIEVPMGITLREIVEEMGGGVPGANNVKAIQTGGPSGGCIPDRELDTSVDYDSLRELGSMMGSGGMVVMDDDTSMVQVAQFYMEFCRGETCGKCIPCRAGTVQMYQLLTKLLDEKATKRDLDRLKELCGMVQATSLCGLGQTAPNPVLSTLHYFEQEYLDLLQPDPVAMANGRG

>Phormidium lacuna HoxU

MSVVTLKINNIDVAAEAGKTVLEAAREAGIRIPTLCHLDGVSDVGACRLCLIEIKGIPKLLPACVTEVSEGMDVTTHTPQLDEYRRMTVEMLFSEGNHVCAVCVANENCELQDVAIEVGMDHSRFTYRFPERGVDISHPQFGIDHNRCILCTRCVRVCDEIEGAHVWDVAGRGAAAKVVTGLNQPWGDVEACTACGKCVDACPTGSIFRKGSTASELDRDRGKLEFLVKAREENQWTR

>Phormidium lacuna HoxY

MDTVNKKVKLATIWLAGCSGCHMSFLDLDEWLFELAKFADMVYSPVGSDIKDYPEDVDVCLVEGAVANEENLELLYKVRQRTKFVISFGDCAVTANVPAMRNMLGSSEPVLKRCYLELGDNTAQLPHEPGIVPELLDRVRPIHELVDIDLFIPGCPPSAPRIQAAIEPLLKGEQPVMEGRSMIKFG

>Phormidium lacuna HoxH

MSKTVVIDPVTRIEGHAKISIFLNDAGDVDDARFHVVEFRGFEKFCEGRPMFEMAGITARICGICPVSHLLAAAKTGDKILAVQVPPAGEKLRRLMNLAQIIQSHTLSFFHLSSPDFLLGWDSDPAKRNVFGLMDANPDLARAGIRLRQFGQTVIELLGAKKIHAAWAVPGGVRSPLSEEGLQWIRDRLPESRQTIETALGLFKQLLDDTLKDEVNIFGQFDSLFMSLVAPDGTWEHYGGHIRFVDSQGNIVADGLREEDYNDFLAEAVEPWSYLKFPYYKPMGYPDGMYRVGPLARLNVCDRMGTPDADRELQEFRQRAGGRCATSSFMYHYARLLEVLACIERIEQYVDDPDLLSSRCRSKAEINNLEGVGVSEAPRGTLFHHYNVDENGLIEKVNLIIATGQNNLAMNKTVTQIAQHYIHNNDVAEGFLNRVEAGIRNFDPCLSCSTHAVGQMPLHIDLIAPDKTVVNTIYRD

>Phormidium lacuna PsaA

MTISPQERDAKVKVEVDKNPVPTSFEKWGKPGHFDRTLARGPKTTTWIWNLHADAHDFDSQTNDLEEVSRKIFSAHFGQLAVIFIWLSGAYFHGARFSNYEAWLTDPTGIKPSAQVVWSIVGQDILNADVGGGFHGIQITSGLFQLWRASGITNEFQLYCTAIGGLVMAALMLFAGWFHYHVSAPKLEWFQNVESMMNHHLAGLLGLGSLGWAGHQIHVSLPINKLLDAGVAPQDIPLPHEFILDTSKMAELYPSFAKGLTPFFTLNWGEYADFLTFKGGLNPQTGGLWLSDTAHHHLAIAVLFIIAGHMYRTNWGIGHSMKEILEAHKGPFTGEGHKGLYEILTTSWHAQLAINLAMLGSLSIIVAHHMYSMPPYPYIATDYPTQLSLFTHHMWIGGFLIVGAGAHAAIFMVRDYDPAKNVDNLLDRVIRHRDAIISHLNWVCIFLGFHSFGLYVHNDTMRAFGRPQDMFSDTGIQLQPIFAQWVQHLHTLAPGNTAPNALASVSPAFGGDVVAVGGKVAMMPIELGTADFLVHHIHAFTIHVTVLILLKGVLFARSSRLIPDKAELGFRFACDGPGRGGTCQVSGWDHVFLGLFWMYNSLSIVIFHFSWKMQSDVWGTVGADGSVSHITYGNFAQSAITINGWLRDFLWAQASQVIGSYGSALSAYGLLFLGAHFVWAFSLMFLFSGRGYWQELIESIVWAHNKLKVAPAIQPRALSIIQGRAVGVAHYLLGGIATTWAFFLARIISVG

>Phormidium lacuna PsaC

MSHSVKIYDTCIGCTQCVRACPLDVLEMVPWDGCKAGQIATSPRTEDCIGCKRCETACPTDFLSVRVYLGAETTRSMGLAY

>Phormidium lacuna PsaB

MATKFPKFSQDLAQDPTTRRIWYGIATSHDFESHDGMTEENLYQKIFASHFGHLAIIFLWTSGNLFHVAWQGNFEQWIKDPLNIRPIAHAIWDPQFGQGAVDAFTQAGASNPVNIAYSGVYHWWYTIGMRTNNDLYQGSIFLLVLAAVMLFAGWLHLQPKYRPSLSWFKNAESRLNHHLAGLFGVSSLAWTGHLVHVAIPESRGQHVGWDNFLSTMPHPAGLKPFFTGNWGVYAQSPDTASHVFGTSEGAGTAILTFLGGFHPQTESLWLTDMAHHHLAIAVLFIVAGHMYRTNFGIGHSIKEILGAHNPPKGTPFGGMLGEGHKGLYDTINNSLHFQLGLALASLGVITSLVAQHMYALPPYAFMAKDYTTMAALYTHHQYIAGFLMVGAFAHGAIFFVRDYDPEMNKNNVLARMLDHKEALISHLSWVSLFLGFHTLGLYVHNDVVVAFGTPEKQILIEPVFAQWVQAASGKALYGFDVLLSNPDSIATTAWPNHANVWLPGWLDAINSGSNSLFLTIGPGDFLVHHAIALGLHTTTLILVKGALDARGSKLMPDKKDFGYSFPCDGPGRGGTCDISAWDAFYLAMFWMLNTLGWLTFYWHWKHLTVWQGNVAQFNESSTYLMGWFRDYLWLNSSQLINGYNPFGVNNLSVWAWMFLFGHLVWATGFMFLISWRGYWQELIETLVWAHERTPLANLVRWKDKPVALSIVQARVVGLAHFTVGYIFTYAAFLIASTAGKFG

>Phormidium lacuna PsaD

MPIKLFNLVQFKLMAEELTGKTPIFGGSTGGLLSAAEREEKYAITWTSSKQQVFEMPTGGAAIMHEGENLLYLARKEQCLALGTQLRTKFKPKLEDFKIYRIFPNGEMEYLHPKDGVFPEKVNEGRPLVNIIDRSIGKNPEPAKLKFSGKQPYTA

>Phormidium lacuna PsaE

MIQRGSKVRILRKESYWYKDLGTVASIDQSGIRYPAIVRFDKVNYNGFSGAAGGVNTNNFALEELEEVAPPAKKKKK

>Phormidium lacuna PsaF

MRRLLALVLTAVLWFSFAPTASADVAGLTPCNESPAFIARAKAATTEQAKQRFELYGRELLCGEEGLPHLIVDGRWSHAGEFLIPGVLFLYIAGWIGWAGRSYLISIRGEKSPEEKEIIIDVPRAISCSLGGVAWPLAAFKEMTTGEMFANDNEIPISPR

>Phormidium lacuna PsaI

MTGDYAASYLPWILIPVVCWLMPVVTMGLLFIYIEKEA

>Phormidium lacuna PsaJ

MKDLQRYLSTAPVLATAWMFITAGILIEFNRIFPDLLFHPLH

>Phormidium lacuna PsaK

MLNSVIFALTPTTSAWSPSIAIVMIVCNILAIAIGKYSIKYPSVGPQAPSPNLFGGFGIPAILATTSFGHILGVGAILGLQTAGVL

>Phormidium lacuna PsaK2

MLFINSTLTLAASSLTTPWSTDVAIIMVASNLFALAIGRYAIQKRGVGPALPISVPGLFEGFGWSELLATASFGHILGAGIILGLGGSGAL

>Phormidium lacuna PsaL

MTTSTSNQMVKPYQGDPQMGHLSTPISDSAFTRTFIGNLPAYRPGLSPLLRGLEIGMAHGYFIGGPWVKLGTQRGTEFANLNGLICGGTLLLIATACLAAYGLVSFQGKQSNSQDSLQSSEGWSQFTAGFFIGGMGSAFLAFFLLENFGAVDAILRGLVNN

>Microcystis aeruginosa HoxY

MSKIRFATVWLAGCSGCHMSFLDLDEWLLELAEKVDVVYSPVGCDLKTYPENVDVCLVEGAIANQDNLELIHLVRQNTKTVVSFGDCAVTANVPAMRNMLGTADPVLKRAYLELGDNTPQLPEEPGIVPELLDQVLPVHQVIPIDIFMPGCPPDADRIRETLIPILKGELPVMAGREMIK

>Microcystis aeruginosa HoxH

MTKTVVIDPVTRIEGHAKISIFLDDGGEVDDVRFHVVEYRGFEKFCEGRPMWEMAGITAR

ICGICPVSHLLCAAKTGDKILAVQIPPAGEKLRRLMNLGQLTQSHALSFFHLSSPDFLLG

WESDPAKRNIFGLIAADPDLARAGIRLRQFGQKVIELLGAKKIHPAWSVPGGVRSPLSEE

GRQWIKERLPESKATLYTALNLFKRLLDDLTIEIAAFGNFPSLFMGLVGKRDEWEHYGGH

IRFTDSQGNIVADNLSEDNYRDYIGESVEKWSYLKFPYYKPLGYPNGIYRVGPLARLNVC

SHFGTEGADIELREYRHRVGGVATSSFYYHYARLVEILGCLERIELLIDDPDIVSPRCRA

EAGINNLQGVGVSEAPRGTLFHDYKVDENGLIETVNLIIATGNNNLAMNQTVKQIAQHYI

HGGEIPEAMLNRVEAGIRCYDPCLSCSTHAMGQMPLQLELVNAAGEVINTRQRG

DNA sequences

>Phormidium lacuna HoxE

ATGACCCCCACCCAGACCTCAACCGCCGCTAAATCATCCCAAAACGCCCCTAAAAAAGACGGCAAGCCCGATAAACGCTTCAAAGTTCTCGACATTACAATGAAGCGTAACCAATATCGACAAGATGCCCTCATCGAAATCCTCCATAAAGCCCAAGAAGCCTTTGGCTTTCTCGAAGAAGATGTTCTAAGTTACATCGCCCATAAACTGCAACTGCCCCTCTCCCAAGTCTACGGCGTTGCCACCTTCTACCATCTGTTCTCCCTCAAACCCAGTGGGGCTCATACCTGCGTCGTCTGTCTGGGAACCGCCTGCTACGTCAAAGGCGGCGGCGACATCCTCAAAGCCGTCGAGGGCGATGTGGGCATCGCCGCCGGGGAAACCACCGAAGACGGGCAAGTCTCCATCGTCACCGCTCGTTGCATTGGTGCTTGTGGGATCGCCCCGGCGGTGGTGTATGACGGCAAAGTCGCCGGCCAACAAACCCCCGAACAAACCTGCGATCGCATCCATAGCTGGAAACAAGAGGACAGCTAA

>Phormidium lacuna HoxF

ATGGAACGAGCTGAACTACTCGAACAGGCCACCGCCGCCAAACAAGCCCAAAAACGCATCCGCATCCACTGTTGCACCTCCACCGGCTGCCAAGCCTCCCAATCCCTCGACGTTAAAAAACAACTCGACGCCGCCATCAAAACCCACCACCTCGGCGACACCGTCGAAGCCGTCGGCGTTGGCTGCATGGGATTCTGTGGACAAGGGCCGATGGTCGAAATCGAAGACACCGAAGCCCCAGCCGAGAAAAAACACTATCAAAAAGTCACCCCCGAACAAGCCGAAAGCATCATCGGCAGCCTCAACGGTTCCGGAGAAGCCGACGCCATTCAAGGCGATCCCAACCACCCCTTCTTTAGCCGTCAACTCCTCATTGTCCGCGAACATAGCGGCCGCATCGATCCCGAACGCATCGACGAATATCTCGCCGTCGGCGGCTACCAAGCCCTACATCACGCCATCTATGAAATGTCCCCGGCTGACGTGGTGCAAGAAATCACCCAATCGGGACTACGGGGACGAGGCGGTGCCGGCTATCCCACGGGCTTAAAATGGGCTACGGTGGCGAAAATGCCCCCCGGACAGAAATACATCATCTGTAACGCCGACGAAGGCGATCCTGGGGCGTTTATGGATCGCAGTGTCTTGGAAAGTGACCCTCATCGCGTCTTAGAAGGGATGGCGATCGCCGGCTACGCCGTGGGCGCAACCCAAGGCTACATCTACGTTCGCGCCGAATATCCCCTCGCCATCACCCGCCTGCAAAAAGCCATCCAACAGGCCAAACGCAAACATTGCCTCGGGGCGCAAATCTTCGACTCCCCCGTAGACTTCACCATCGAAATTCGCGTCGGTGCCGGGGCCTTCGTTTGTGGCGAAGAAACTGCCCTCATCTCCTCCGTCGAAGGCGGACGAGGCAACCCTCGCCCTCGCCCCCCCTATCCCGCTGTCTCCGGCCTTCACGGCTGTCCGACACTCATCAACAACGTAGAAACCCTGGGGAATATCTCCCCCATCATCCGCAACGGGGCCGAGTGGTTCGCCGGCATTGGCACCGAACGCAGTAAAGGCACTAAAATCTTCTCCCTCACCGGCAAAATCCGCAACAACGGACTGATTGAAGTTCCGATGGGGATTACCCTCCGGGAAATTGTCGAAGAGATGGGAGGCGGCGTTCCTGGTGCTAATAACGTCAAAGCCATTCAAACCGGGGGTCCTTCTGGCGGGTGTATTCCCGATCGCGAACTCGACACCTCCGTCGATTATGACTCCCTACGGGAACTCGGGTCCATGATGGGGTCCGGTGGCATGGTGGTCATGGACGACGACACCAGCATGGTGCAAGTGGCCCAGTTCTACATGGAGTTTTGCCGAGGCGAAACCTGTGGTAAGTGCATCCCCTGTCGCGCCGGAACTGTGCAGATGTATCAACTTCTCACCAAACTCCTAGACGAGAAAGCCACGAAACGGGATTTAGATCGACTCAAAGAACTCTGCGGCATGGTGCAAGCCACCAGTCTCTGTGGTTTAGGCCAAACCGCCCCCAACCCAGTACTCAGCACCCTCCATTACTTTGAACAAGAGTACCTAGACCTATTACAACCCGATCCTGTGGCGATGGCCAACGGTCGAGGTTAG

>Phormidium lacuna HoxU

ATGTCTGTCGTCACCCTCAAAATCAACAATATCGATGTCGCGGCCGAAGCCGGTAAAACCGTCCTCGAAGCCGCACGCGAGGCGGGAATCCGCATTCCCACCCTTTGCCATCTCGATGGTGTCTCCGATGTGGGCGCTTGTCGCCTCTGTTTAATCGAAATTAAAGGGATTCCTAAACTCCTCCCCGCCTGCGTCACGGAAGTGAGCGAGGGGATGGACGTCACCACCCACACCCCCCAGTTGGACGAGTATCGCCGCATGACCGTGGAAATGCTCTTTTCCGAAGGAAATCATGTTTGCGCCGTCTGTGTCGCCAACGAGAACTGTGAATTGCAAGATGTGGCCATCGAAGTGGGGATGGATCACAGTCGCTTTACCTATCGCTTCCCGGAACGGGGAGTCGATATCTCCCACCCCCAATTTGGCATCGACCATAACCGCTGTATCCTCTGTACCCGCTGTGTGCGCGTCTGTGATGAAATCGAAGGCGCTCATGTGTGGGATGTGGCCGGACGCGGGGCCGCCGCGAAGGTGGTGACGGGGTTGAATCAACCCTGGGGCGATGTGGAGGCCTGTACCGCTTGCGGGAAATGCGTTGATGCTTGTCCCACGGGGTCGATTTTCCGTAAGGGAAGTACGGCTTCGGAACTCGATCGCGATCGCGGCAAACTGGAATTTTTAGTCAAAGCACGGGAAGAAAATCAATGGACACGGTAA

>Phormidium lacuna HoxY

ATGGACACGGTAAACAAGAAAGTAAAACTGGCAACGATTTGGCTGGCCGGCTGTTCCGGTTGTCATATGTCCTTCCTGGACTTGGACGAATGGCTGTTTGAACTGGCCAAGTTCGCCGACATGGTCTATAGTCCCGTCGGTTCGGATATCAAGGACTATCCTGAGGATGTGGATGTCTGTCTGGTGGAGGGGGCTGTGGCCAATGAGGAAAACCTCGAACTGTTGTACAAAGTTCGCCAACGCACCAAGTTTGTCATCTCCTTCGGCGATTGTGCGGTGACGGCCAATGTCCCCGCCATGCGCAATATGTTGGGCAGTTCTGAGCCAGTTCTCAAACGCTGCTACCTCGAACTCGGGGACAATACCGCTCAACTTCCCCATGAACCGGGGATTGTCCCGGAACTGCTCGATCGCGTCCGTCCGATTCATGAATTAGTAGATATTGACCTGTTTATCCCCGGCTGTCCCCCCTCCGCCCCTCGCATCCAAGCTGCGATCGAACCGCTACTCAAGGGAGAACAGCCTGTCATGGAAGGGCGATCGATGATTAAGTTCGGCTAA

>Phormidium lacuna HoxH

ATGAGTAAAACTGTTGTTATTGATCCCGTCACTCGCATTGAGGGTCACGCCAAAATTTCCATCTTTCTCAACGATGCTGGAGATGTCGATGACGCTCGTTTCCATGTCGTTGAATTTCGCGGTTTTGAGAAATTCTGCGAAGGTCGTCCCATGTTTGAGATGGCCGGAATTACCGCCCGGATTTGTGGAATTTGTCCGGTTAGCCATCTTTTGGCGGCGGCGAAAACCGGCGATAAAATCCTAGCGGTGCAAGTTCCCCCGGCTGGGGAGAAACTGCGACGGTTGATGAATTTAGCCCAAATCATCCAATCTCACACCCTGAGTTTCTTCCATCTCAGTAGTCCCGATTTTCTCCTAGGCTGGGATAGTGACCCCGCCAAACGCAATGTCTTCGGCTTGATGGACGCGAATCCCGACTTAGCCCGGGCCGGGATTCGTCTGCGTCAGTTTGGACAGACGGTGATTGAACTGTTGGGGGCTAAGAAAATCCATGCGGCTTGGGCGGTTCCGGGTGGGGTGCGATCGCCCCTGTCGGAAGAGGGCCTACAATGGATTCGCGATCGCCTACCCGAATCCCGCCAAACCATCGAAACGGCCCTGGGCCTGTTCAAACAACTCCTCGACGACACCCTAAAAGATGAAGTTAACATCTTTGGGCAGTTTGACTCCCTCTTTATGAGTCTCGTCGCCCCCGATGGCACTTGGGAACATTACGGCGGTCATATCCGCTTCGTCGATAGCCAAGGGAACATCGTCGCCGATGGCTTGCGGGAAGAAGACTATAACGACTTCCTAGCCGAAGCCGTTGAACCCTGGTCTTATCTCAAATTCCCCTATTACAAACCCATGGGCTATCCTGATGGGATGTATCGGGTGGGTCCTTTGGCCCGGTTAAATGTCTGCGATCGCATGGGAACCCCCGACGCCGATCGCGAACTGCAAGAATTTCGCCAACGGGCCGGTGGCCGCTGCGCCACCTCCTCCTTCATGTACCACTACGCCCGTCTCCTAGAAGTTCTCGCCTGTATCGAACGCATTGAACAGTATGTCGATGACCCCGATTTATTGTCCTCCCGTTGTCGGTCTAAAGCCGAAATTAACAACCTCGAAGGAGTCGGCGTGAGCGAAGCCCCTCGCGGAACTCTATTTCATCATTACAATGTAGACGAGAACGGTCTGATTGAGAAAGTTAACCTCATCATCGCCACGGGACAAAACAACCTGGCCATGAACAAAACCGTCACCCAGATTGCCCAACATTACATCCATAACAACGATGTGGCTGAGGGCTTTCTCAACCGAGTTGAAGCCGGAATCCGCAACTTCGACCCCTGTCTCAGTTGTTCAACCCACGCCGTCGGCCAAATGCCCCTCCATATCGACCTCATCGCCCCCGACAAAACCGTCGTCAACACCATCTACCGCGACTAA

>Phormidium lacuna PsaA

ATGACGATTAGTCCCCAGGAGCGGGATGCAAAAGTCAAAGTCGAGGTCGACAAGAACCCGGTCCCAACTTCTTTCGAGAAGTGGGGCAAACCCGGTCACTTCGATCGCACTTTGGCACGAGGTCCAAAAACCACCACTTGGATTTGGAACCTCCATGCAGACGCTCATGATTTCGACAGTCAAACCAACGACTTAGAAGAAGTATCGCGCAAAATCTTTAGCGCCCACTTCGGTCAACTGGCAGTCATTTTTATTTGGTTAAGCGGTGCCTACTTCCACGGCGCGCGTTTTTCCAACTATGAAGCTTGGTTAACCGATCCTACCGGGATCAAACCGAGCGCCCAAGTAGTCTGGTCCATCGTGGGTCAAGACATCCTCAACGCCGACGTTGGTGGTGGCTTCCACGGGATTCAAATTACCTCTGGCTTATTCCAACTCTGGCGTGCCAGCGGCATTACCAATGAGTTCCAGCTATACTGCACCGCCATCGGTGGTCTAGTCATGGCAGCTCTGATGCTGTTCGCCGGTTGGTTCCATTACCACGTCAGTGCACCGAAACTGGAATGGTTCCAAAACGTGGAATCGATGATGAACCACCACCTGGCTGGTTTACTCGGACTTGGCTCCTTAGGTTGGGCCGGTCACCAAATCCACGTTTCGCTTCCCATTAATAAACTTCTGGATGCTGGTGTTGCACCGCAGGATATTCCCCTGCCGCACGAGTTTATCCTTGATACAAGCAAAATGGCGGAACTGTATCCCAGTTTTGCCAAAGGTTTAACCCCATTCTTTACCTTAAATTGGGGTGAATATGCAGACTTCCTAACCTTCAAAGGTGGCTTGAACCCCCAAACCGGTGGTCTCTGGCTGAGCGACACGGCACACCATCACTTGGCGATCGCCGTCTTGTTCATCATCGCCGGTCACATGTACCGAACCAACTGGGGTATCGGTCACAGCATGAAGGAAATCCTGGAAGCTCACAAAGGACCCTTCACCGGAGAAGGTCACAAAGGACTCTATGAGATCCTAACCACCTCCTGGCACGCTCAACTCGCGATTAACCTTGCGATGCTGGGTTCCCTGAGTATCATCGTGGCTCACCACATGTACTCCATGCCTCCCTATCCGTACATCGCGACGGATTACCCGACACAACTGTCGCTGTTCACCCACCATATGTGGATTGGCGGCTTCCTGATTGTCGGTGCAGGCGCTCACGCTGCCATCTTTATGGTTCGTGACTACGACCCTGCCAAAAACGTGGATAACCTGCTCGATCGGGTCATCCGCCATCGAGATGCAATCATTTCTCACCTGAACTGGGTTTGTATCTTCTTGGGCTTCCACAGCTTTGGACTCTACGTCCATAACGACACCATGCGTGCCTTTGGTCGTCCTCAAGATATGTTCTCCGACACGGGGATCCAGCTTCAACCGATTTTTGCCCAGTGGGTTCAACACTTACACACCTTGGCACCGGGAAATACAGCTCCCAATGCCCTGGCCAGTGTGAGTCCTGCCTTTGGCGGCGATGTCGTCGCTGTCGGTGGAAAAGTCGCCATGATGCCGATTGAACTCGGTACAGCCGACTTCCTGGTTCACCACATTCACGCGTTCACGATTCACGTGACCGTGCTGATTCTACTCAAAGGCGTACTGTTTGCTCGCAGCTCTCGCCTGATTCCCGATAAAGCCGAGCTAGGCTTCCGCTTTGCTTGCGACGGTCCGGGACGTGGCGGTACCTGCCAAGTCTCTGGTTGGGACCATGTGTTCTTGGGTCTGTTCTGGATGTACAACAGCCTCTCCATCGTCATCTTCCACTTCAGTTGGAAAATGCAATCCGATGTTTGGGGAACAGTTGGTGCAGACGGTTCGGTGTCCCACATCACCTATGGCAACTTTGCCCAAAGCGCCATTACCATTAATGGGTGGTTGCGCGACTTCCTGTGGGCGCAAGCCTCTCAGGTAATCGGTTCCTACGGTTCAGCCTTGTCCGCCTATGGACTGCTGTTCCTCGGTGCTCACTTCGTTTGGGCATTCAGCCTCATGTTCCTCTTTAGTGGACGTGGCTACTGGCAAGAGCTAATTGAGTCGATTGTTTGGGCTCATAACAAGCTCAAAGTCGCTCCCGCGATTCAACCCCGCGCCCTGAGCATTATTCAAGGTCGGGCCGTTGGTGTCGCCCACTATCTGTTAGGTGGGATTGCCACAACCTGGGCATTCTTCCTGGCACGGATCATTTCAGTAGGATGA

>Phormidium lacuna PsaB

ATGGCAACGAAATTCCCAAAATTTAGCCAAGACTTGGCACAGGATCCAACCACACGGCGGATCTGGTATGGGATTGCTACGTCTCACGATTTCGAAAGCCATGATGGTATGACGGAAGAAAATCTTTACCAAAAGATTTTCGCCTCCCACTTCGGACACCTCGCCATCATCTTTCTGTGGACCTCGGGCAACCTCTTCCATGTCGCTTGGCAAGGCAACTTCGAGCAGTGGATCAAAGATCCCCTCAACATCCGTCCCATCGCCCACGCGATTTGGGATCCTCAATTCGGTCAAGGCGCTGTTGATGCCTTCACCCAAGCCGGGGCATCGAACCCGGTCAACATCGCGTACTCCGGCGTGTACCACTGGTGGTACACCATCGGAATGCGCACCAACAATGACCTTTACCAAGGGTCGATCTTCCTTCTGGTTCTGGCGGCTGTCATGCTCTTCGCGGGCTGGCTACACCTGCAACCGAAATATCGTCCGAGTCTGTCTTGGTTCAAAAATGCTGAGTCTCGCCTGAACCACCACCTCGCGGGTCTGTTCGGAGTTAGCTCACTGGCTTGGACGGGTCACCTGGTGCATGTCGCCATCCCTGAATCTCGGGGTCAGCATGTGGGTTGGGATAACTTCCTCAGCACCATGCCTCACCCGGCCGGACTGAAGCCTTTCTTCACCGGTAACTGGGGCGTCTATGCTCAGAGTCCTGACACCGCAAGCCATGTCTTCGGCACGTCCGAAGGTGCAGGAACCGCTATCTTGACCTTCTTGGGTGGCTTCCACCCGCAAACCGAGTCCCTGTGGCTGACGGATATGGCGCATCACCATCTGGCGATCGCCGTCCTGTTCATCGTCGCAGGTCACATGTACCGCACCAACTTTGGTATCGGTCACAGCATTAAAGAGATCCTCGGCGCTCACAATCCCCCCAAAGGCACTCCTTTCGGCGGAATGTTGGGTGAAGGTCACAAAGGTCTCTACGACACCATCAACAACTCGCTGCACTTCCAACTGGGTCTGGCTCTCGCCTCCTTAGGTGTGATTACCTCCCTGGTCGCGCAGCACATGTACGCTCTGCCTCCCTACGCCTTCATGGCGAAGGACTATACCACCATGGCAGCGTTGTACACCCATCACCAGTACATTGCTGGCTTCCTGATGGTTGGGGCATTTGCTCACGGCGCAATCTTCTTTGTCCGTGACTATGATCCCGAAATGAACAAAAACAATGTTCTGGCTCGGATGCTGGATCATAAAGAGGCTCTCATTTCTCACCTGAGCTGGGTGTCTCTGTTCCTCGGTTTCCATACCTTGGGACTCTATGTTCACAACGACGTTGTCGTTGCTTTCGGTACCCCCGAAAAACAAATCCTGATTGAGCCGGTCTTCGCCCAATGGGTTCAGGCCGCATCGGGTAAAGCCCTCTACGGCTTTGATGTTCTTCTGTCGAACCCCGACAGCATTGCCACCACCGCTTGGCCCAACCATGCCAACGTGTGGCTACCCGGTTGGTTGGATGCCATCAACAGTGGTTCCAACTCCCTCTTCTTGACCATTGGTCCTGGTGACTTCCTAGTCCACCATGCGATCGCCCTCGGTCTGCATACCACCACCTTGATTCTCGTTAAAGGTGCTTTGGATGCCCGTGGTTCTAAGCTGATGCCGGACAAAAAAGACTTCGGTTACAGCTTCCCTTGTGATGGTCCTGGTCGTGGCGGTACGTGCGACATCTCTGCTTGGGATGCCTTCTATCTCGCCATGTTCTGGATGCTGAACACCTTAGGTTGGTTGACCTTCTACTGGCACTGGAAACACCTCACCGTTTGGCAAGGTAACGTTGCCCAGTTCAATGAGTCGTCCACCTATCTGATGGGTTGGTTCCGCGATTACCTGTGGTTGAACTCGTCCCAGTTAATCAACGGCTACAATCCCTTCGGTGTCAATAACCTCTCGGTTTGGGCTTGGATGTTCCTGTTCGGTCACCTGGTTTGGGCGACAGGCTTCATGTTCCTCATCTCTTGGCGGGGTTACTGGCAAGAACTCATCGAAACCTTGGTTTGGGCACACGAGCGCACTCCTCTGGCGAACCTGGTTCGTTGGAAAGACAAGCCCGTTGCACTCTCCATCGTTCAGGCTCGGGTTGTGGGTCTAGCTCACTTTACTGTGGGGTATATCTTCACCTACGCGGCCTTCCTGATTGCCTCAACGGCTGGTAAGTTCGGTTAA

>Phormidium lacuna PsaC

ATGTCTCATTCGGTAAAAATCTATGACACCTGCATCGGATGCACTCAATGCGTCCGTGCTTGTCCCTTAGACGTGTTGGAAATGGTGCCTTGGGATGGCTGTAAAGCCGGCCAAATCGCCACCTCTCCCCGTACGGAAGACTGCATCGGCTGCAAACGTTGCGAAACGGCTTGCCCGACCGACTTCTTGAGCGTCCGAGTCTATCTTGGTGCGGAAACCACCCGCAGCATGGGTCTGGCTTACTAA

>Phormidium lacuna PsaD

ATGCCCATCAAGCTCTTTAATCTCGTTCAATTTAAACTTATGGCAGAAGAACTGACTGGAAAAACTCCGATTTTCGGTGGTAGCACCGGTGGTTTGCTTTCGGCAGCCGAACGTGAAGAAAAGTATGCCATCACCTGGACTAGCTCCAAGCAGCAAGTGTTTGAGATGCCCACCGGCGGTGCGGCGATCATGCACGAAGGCGAAAACCTGCTGTACTTGGCTCGCAAGGAACAGTGTTTGGCCCTCGGAACCCAACTGCGGACGAAGTTCAAGCCGAAACTCGAAGATTTTAAAATCTATCGGATTTTCCCCAACGGTGAAATGGAATATCTCCATCCCAAAGATGGTGTGTTCCCCGAGAAAGTGAACGAAGGTCGTCCTTTGGTCAACATCATCGATCGCAGCATCGGCAAAAACCCCGAACCCGCTAAACTCAAGTTCAGCGGCAAACAACCCTACACCGCTTAA

>Phormidium lacuna PsaE

ATGATTCAACGTGGTTCTAAAGTGCGGATTCTCCGTAAAGAATCCTATTGGTATAAAGATCTCGGCACGGTCGCGAGTATTGACCAAAGCGGAATCCGCTACCCGGCGATCGTTCGCTTCGACAAAGTGAACTACAACGGTTTCAGCGGTGCAGCCGGTGGTGTAAACACCAATAACTTCGCTCTCGAAGAATTGGAGGAAGTTGCTCCCCCCGCCAAGAAGAAGAAAAAATAA

>Phormidium lacuna PsaF

ATGCGACGACTATTAGCGCTGGTACTGACAGCAGTTCTTTGGTTCAGCTTTGCTCCCACCGCTTCGGCTGACGTGGCGGGTCTGACTCCCTGTAACGAGAGTCCTGCCTTTATCGCCCGTGCCAAAGCCGCAACCACTGAGCAAGCGAAACAACGGTTTGAACTGTATGGACGCGAACTTCTCTGCGGAGAAGAGGGCCTTCCTCACCTGATTGTGGATGGTCGCTGGAGCCATGCTGGAGAATTTCTCATTCCTGGCGTGCTGTTTCTGTACATTGCTGGCTGGATTGGCTGGGCCGGTCGGAGTTATCTAATTTCTATTCGTGGCGAGAAATCTCCCGAAGAAAAAGAAATCATCATCGACGTTCCTCGGGCTATTAGCTGCTCCTTAGGTGGCGTTGCTTGGCCTCTAGCGGCATTCAAAGAAATGACCACTGGAGAAATGTTCGCCAACGACAATGAAATCCCCATTTCTCCCCGCTAG

>Phormidium lacuna PsaI

ATGACTGGTGATTACGCTGCTTCCTATCTTCCCTGGATTCTGATTCCGGTTGTCTGCTGGCTGATGCCGGTCGTCACCATGGGTCTGCTATTTATTTACATCGAAAAAGAAGCTTAG

>Phormidium lacuna PsaJ

ATGAAAGACCTACAAAGATACCTGTCAACCGCTCCCGTTTTGGCAACGGCCTGGATGTTTATTACGGCGGGAATTTTGATTGAGTTTAATCGCATTTTTCCCGATTTACTCTTCCATCCTCTTCACTAA

>Phormidium lacuna PsaK

ATGCTCAACAGCGTTATCTTCGCTCTGACCCCCACCACGTCAGCTTGGAGTCCCAGCATTGCCATTGTCATGATTGTCTGCAACATTCTGGCCATTGCCATTGGTAAATACTCAATTAAATATCCCAGTGTTGGCCCTCAAGCTCCCTCGCCGAATCTCTTTGGTGGCTTTGGCATTCCTGCCATTCTGGCTACCACCAGTTTTGGGCATATTCTAGGTGTTGGGGCGATTTTAGGCTTACAAACCGCAGGTGTCCTTTAA

>Phormidium lacuna PsaK2

ATGCTTTTTATCAACAGCACCTTAACTCTAGCGGCCTCGAGCTTAACCACTCCCTGGTCAACCGACGTTGCCATCATTATGGTTGCGTCTAACCTATTTGCCCTGGCCATCGGACGCTATGCCATTCAAAAACGAGGGGTTGGCCCAGCATTGCCGATCTCGGTTCCAGGGCTGTTTGAGGGCTTTGGTTGGTCAGAACTTCTGGCGACGGCGAGCTTTGGCCATATCCTCGGTGCCGGTATTATCCTGGGTCTAGGTGGTTCTGGGGCCTTATAG

>Phormidium lacuna PsaL

ATGACGACTTCCACCAGCAATCAGATGGTGAAGCCTTATCAGGGCGACCCTCAGATGGGTCATCTGTCCACCCCCATTAGCGACTCTGCCTTTACCCGCACCTTTATCGGCAACCTTCCCGCCTACCGGCCCGGGTTGTCCCCCCTGCTGCGCGGACTTGAGATCGGCATGGCTCACGGCTACTTCATCGGCGGCCCCTGGGTCAAACTCGGAACCCAACGCGGTACCGAGTTCGCCAACCTCAACGGCTTGATTTGTGGCGGAACCCTGTTGCTCATTGCAACGGCCTGTTTAGCCGCTTATGGATTAGTCAGCTTCCAAGGCAAACAATCCAACAGCCAAGATTCCCTGCAAAGTTCCGAGGGCTGGAGTCAGTTCACGGCTGGATTTTTCATCGGTGGCATGGGAAGTGCATTCTTGGCATTTTTCTTGTTAGAAAACTTTGGTGCTGTTGATGCCATTCTTCGTGGATTGGTTAACAACTAA

>Microcystis aeruginosa HoxY

ATGTCTAAAATTCGCTTTGCTACTGTCTGGTTAGCCGGTTGTTCGGGATGCCATATGTCCTTCCTCGATTTGGACGAATGGCTGTTAGAATTAGCCGAAAAAGTTGACGTGGTGTATAGTCCCGTCGGCTGCGATCTGAAAACCTATCCGGAAAATGTCGATGTCTGTTTAGTGGAAGGAGCGATCGCTAATCAGGATAATTTAGAACTAATTCATCTAGTCCGTCAAAATACCAAAACCGTCGTTTCTTTTGGTGATTGCGCCGTGACTGCTAACGTTCCCGCTATGCGGAATATGTTAGGAACTGCCGAACCCGTGCTAAAAAGAGCTTATTTAGAGTTAGGAGATAACACCCCTCAACTACCAGAAGAACCCGGTATCGTTCCCGAATTATTAGATCAAGTGCTGCCCGTCCATCAAGTCATTCCCATCGACATTTTTATGCCGGGTTGTCCCCCCGATGCGGACAGAATTCGCGAGACATTGATTCCCATTTAAAAGGGGAATTACCGGTGATGGCAGGACGAGAAATGATTAAATTTGGTTAA

>Microcystis aeruginosa HoxH

ATGACTAAAACCGTCGTTATCGATCCCGTCACCCGGATCGAAGGCCATGCCAAGATCTCGATTTTCCTTGACGATGGGGGGGAAGTGGATGACGTGCGTTTTCATGTGGTCGAGTATCGTGGTTTTGAGAAATTCTGCGAGGGACGGCCGATGTGGGAGATGGCGGGAATTACTGCCCGAATTTGCGGCATTTGCCCCGTTAGTCACCTACTTTGTGCCGCCAAAACCGGCGATAAAATTCTAGCGGTGCAAATCCCCCCTGCGGGGGAAAAACTGCGGCGCTTGATGAATTTGGGACAATTAACCCAATCCCATGCTTTAAGCTTTTTTCATCTTAGCAGTCCCGATTTTCTGCTTGGTTGGGAAAGTGACCCCGCTAAACGGAATGTTTTCGGTTTAATTGCGGCCGATCCCGACCTAGCGCGAGCAGGCATCCGTTTACGTCAATTTGGACAGAAAGTTATTGAACTTTTGGGAGCAAAAAAAATTCACCCCGCTTGGTCTGTCCCGGGGGGTGTGCGTTCCCCTTTAAGCGAAGAAGGGCGACAATGGATTAAAGAACGACTGCCGGAGTCTAAAGCGACTCTTTACACGGCTTTAAACCTATTTAAAGGACTTCTGGACAATTTAACCACAGAAATCGCCGCTTTTGGTAATTTCCCTTCTCTGTTTATGGGATTGGTTGGTAAACGGGACGAGTGGGAACACTACGGTGGTCATATTCGTTTTACCGATAGTCAAGGCAATATCGTAGCCGATAACCTGAGCGAAGACAATTATCGCGATTATATCGGCGAATCAGTGGAAAAATGGTCTTATTTGAAGTTTCCCTACTATAAACCCCTCGGTTATCCCAATGGTATCTATCGAGTTGGTCCCTTGGCCCGCTTAAATGTTTGTTCCCATTTTGGCACGGAGGGAGCGGATATCGAGTTACGCGAATATCGCCATCGGGTCGGTGGTGTAGCAACCTCTTCTTTTTATTATCATTATGCTCGTTTAGTCGAGATTTTGGCTTGTTTAGAACAGATTGAACGTTTAATCGATGATCCCGATATAGTTTCTCAACGCTGCCGGGCCGAAGCAGGAATTAATAATCTGCAAGGGGTGGGAGTTAGTGAAGCGCCCCGGGGTACTTTATTCCATGATTATAAAGTGGATGAAAATGGTTTGATTGAAACGGTGAATTTAATTATTGCCACGGGTAATAATAATTTGGCCATGAATCAAACGGTGAAACAAATCGCCCAACATTATATCCATGGTGGCGAAATTCCCGAAGCGATGTTAAACCGAGTGGAAGCGGGTATTCGTTGCTATGATCCCTGTTTAAGTTGTTCCACTCACGCTATGGGACAAATGCCGCTACAGCTAGAGTTAGTTAATGCGGCCGGTGAGGTGATTAATACTCGACAAAGGGGTTAA
